# Why Are Left-Handed G-Quadruplexes Scarce?

**DOI:** 10.1021/acs.jpclett.3c03589

**Published:** 2024-03-13

**Authors:** Michał Jurkowski, Mateusz Kogut, Subrahmanyam Sappati, Jacek Czub

**Affiliations:** † Department of Physical Chemistry, Gdańsk University of Technology, Narutowicza Street 11/12, 80-233 Gdańsk, Poland; ‡ BioTechMed Center, Gdańsk University of Technology, Narutowicza Street 11/12, 80-233 Gdańsk, Poland

## Abstract

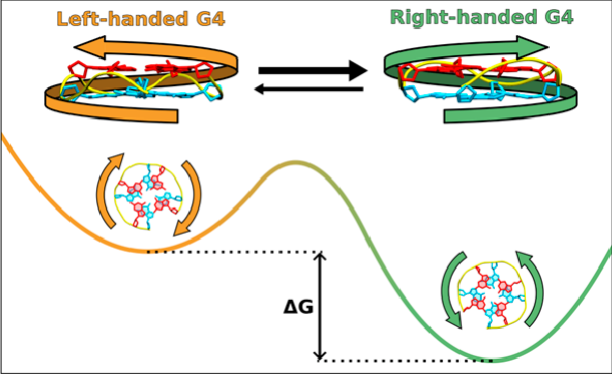

G-quadruplexes (G4s) are nucleic acid structures crucial for the regulation of gene expression and genome maintenance. While they hold promise as nanodevice components, achieving desired G4 folds requires understanding the interplay between stability and structural properties, like helicity. Although right-handed G4 structures dominate the experimental data, the molecular basis for this preference over left-handed helicity is unclear. To address this, we employ all-atom molecular dynamics simulations and quantum chemical methods. Our results reveal that right-handed G4s exhibit greater thermodynamic and kinetic stability as a result of favorable sugar–phosphate backbone conformations in guanine tracts. Moreover, while hydrogen-bonding patterns influence helicity-specific G4 loop conformations, they minimally affect stability differences. We also elucidate the strong correlation between helicity and the strand progression direction, essential for G4 structures. These findings deepen our understanding of G4s, providing molecular-level insights into their structural and energetic preferences, which could inform the design of novel nanodevices.

G-quadruplexes (G4s) are four-stranded structures of nucleic acids formed by sequences containing guanine tracts (G-tracts), in which guanine bases associate through Hoogsteen-type hydrogen bonds into planar tetrads (G-tetrads), additionally stabilized by monovalent cations.^1,2^

The human genome contains more than 700 000 potential G4-forming motifs,^3^ out of which ∼120 000 were observed to form stable G4 structures in cells in a recent genome-wide ChIP-Seq assay.^4^ In the same study, G4 structures were found in more than 60% of promoters (especially at the transcription start site) and ∼70% of genes.^4^ DNA G4s also form in other important genomic regions, including telomeres, immunoglobulin switch regions, and origins of replication, where they have been shown to play an important role in genome maintenance, regulation of gene expression, and replication.^5−15^ Dependent upon the length and content of intervening sequences, called loops, as well as environmental factors, G4-forming motifs can adopt different backbone topologies, leading to remarkable structural diversity.^16−20^ This polymorphism, on the one hand, makes them versatile and potentially programmable building blocks for nanotechnology but, on the other hand, requires a reliable way of predicting and controlling G4 folding preferences.^21−23^

A fundamental structural feature of G4s is their helicity, as is the case for the DNA double helix. Until recently, all solved G4 structures showed a right-handed (RH) helical twist between the adjacent G-tetrad planes (Figure 1, right). However, in 2015, Phan et al. reported the first nuclear magnetic resonance (NMR) structure of the left-handed G4 formed by the sequence derived from the designed antiproliferative aptamer AS1411,^24^ broadening the space of possible G4 folds.^25^ This structure, denoted as *Z*-G4, consists of two left-handed, two-layered G4 blocks stacked together and connected by the double thymine linker (TT). The first of these blocks is formed by the sequence (TGG)_4_, which by itself folds into the RH parallel-stranded G4 but changes its helicity to LH when coupled to the second block.^26^ The latter non-canonical block contains one discontinued G-tract created by the 5′- and 3′-terminal guanosines and is capable of forming dimers of LH G4s associated by cofacial stacking.

**Figure 1:**
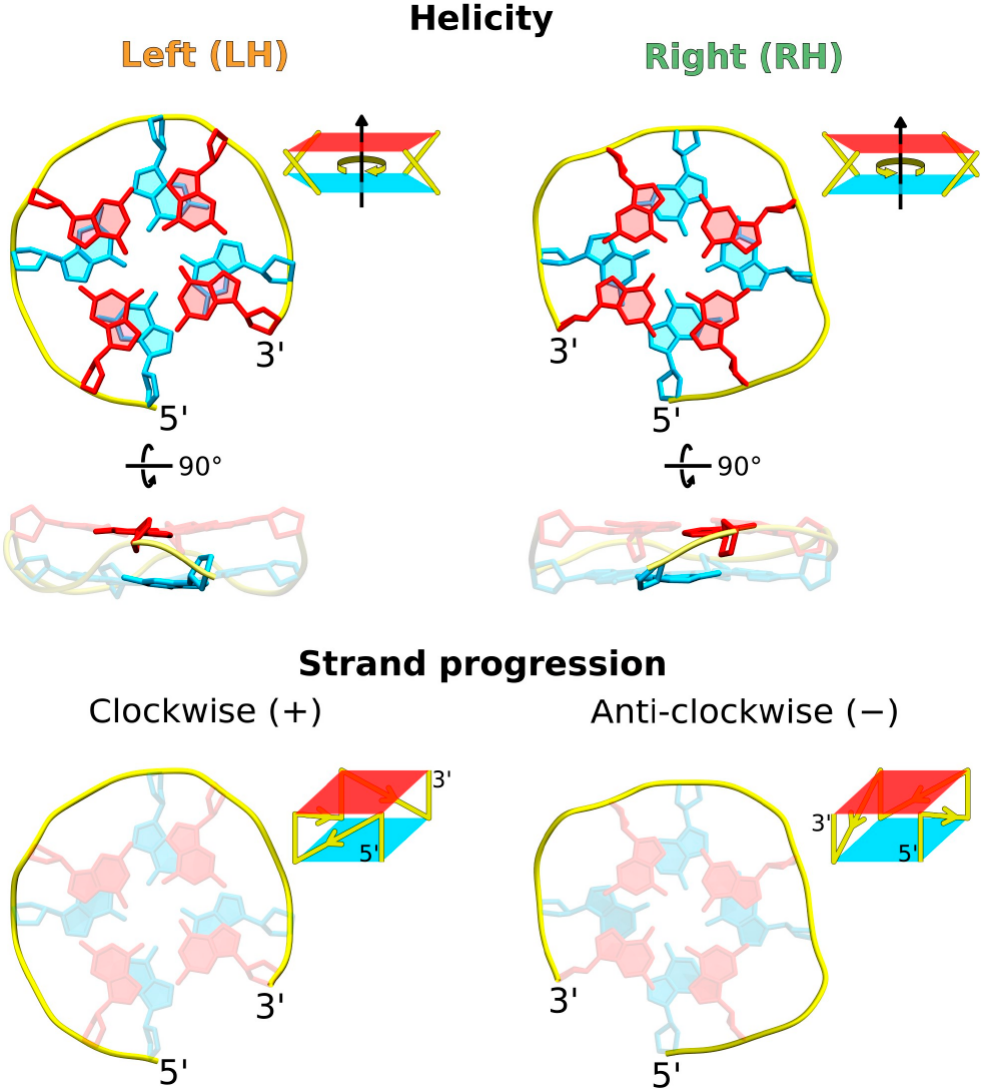
Comparison of the left-handed (LH) and right-handed (RH) helicity in two-layer G4s; 5′- and 3′-terminal G-tetrads are colored cyan and red, respectively. Thus far, all experimentally determined LH G4 structures display the clockwise (+) strand progression, while RH G4 structures exhibit the anti-clockwise (−) direction of strand progression. Note that the opposite direction of strand progression, along with the identical *anti* glycosidic conformation, leads to opposing polarities of the G-tetrads in both cases (Figure S1 of the Supporting Information).

Since then, several other left-handed G4s have been solved (see Table S1 of the Supporting Information), out of which all are parallel-stranded and, similar to their RH counterparts, characterized by the *anti* glycosidic conformation.^26−29^ However, as opposed to parallel RH G4s, LH structures display a clockwise strand progression (Figure 1). Importantly, every left-handed structure observed thus far consists of two parallel G4 blocks stacked on each other (either linked or not), suggesting that adopting the LH helicity might in fact require additional stabilization as a result of cofacial interactions.^30,31^

The need for this stabilization, only a very few solved structures of left-handed G4s, and relatively high sensitivity to sequence changes^26^ suggest that LH G4s are much less stable than their RH counterparts. However, the molecular mechanism behind this difference has yet to be elucidated.

Here, to address this question, we use atomistic molecular dynamics simulations (total sampling time of 370 μs) augmented with quantum chemical calculations. Our findings substantiate and offer a comprehensive molecular-level explanation for the increased stability of the RH G4s. Additionally, we elucidate the reasons behind the distinct directions of strand progression exhibited by the RH and LH G4s.

*Right-Handed Parallel G4s Show Higher Thermodynamic and Kinetic Stability*. To be able to directly address the relation between the helicity and stability of G4s, we first tested to what extent the G4 folding equilibrium is affected by an additional G4 block present in the experimental LH structures. To this end, we computed the free energy profile for the folding of parallel G4s with the GGTGGTGGTGG sequence (T1 sq) and either LH or RH helicity, in both the monomeric and dimeric forms, where the “dimeric” state corresponds to being bound to the fully formed G4 block (see the Methods of the Supporting Information for details). The calculations were performed using GROMACS 2020^32^ combined with the Amber bsc1 force field.^33^ The profiles in Figure 2a clearly show that the monomeric two-layered and parallel G4s are unstable, with their folded state (RoG < 0.37 nm) being ∼6.0 kcal/mol less favorable than the unfolded state (RoG > 0.37 nm), in agreement with the experimental and computational data.^34−36^ However, as anticipated, the presence of the additional block shifts the equilibrium toward the folded state (Figure 2a, bottom), as indicated by the pronounced minima at 0.355 and 0.36 nm (−19.5 and −18 kcal/mol for RH and LH, respectively). It also comes as no surprise that the RH helicity is associated with greater thermodynamic and kinetic stability. Indeed, the computed folding free energy for RH is by 0.8 and 2.8 kcal/mol more favorable than for LH, in the monomeric state (Figure 2a, top) and G4-bound state (Figure 2a, bottom), respectively, while the unfolding barriers are considerably higher for RH G4s, pointing to their longer lifetimes. Given that our simpler monomeric systems capture the crucial stability difference, the following analysis is focused on the comparison of two-layered parallel-stranded G4 monomers with RH and LH helicity.

**Figure 2:**
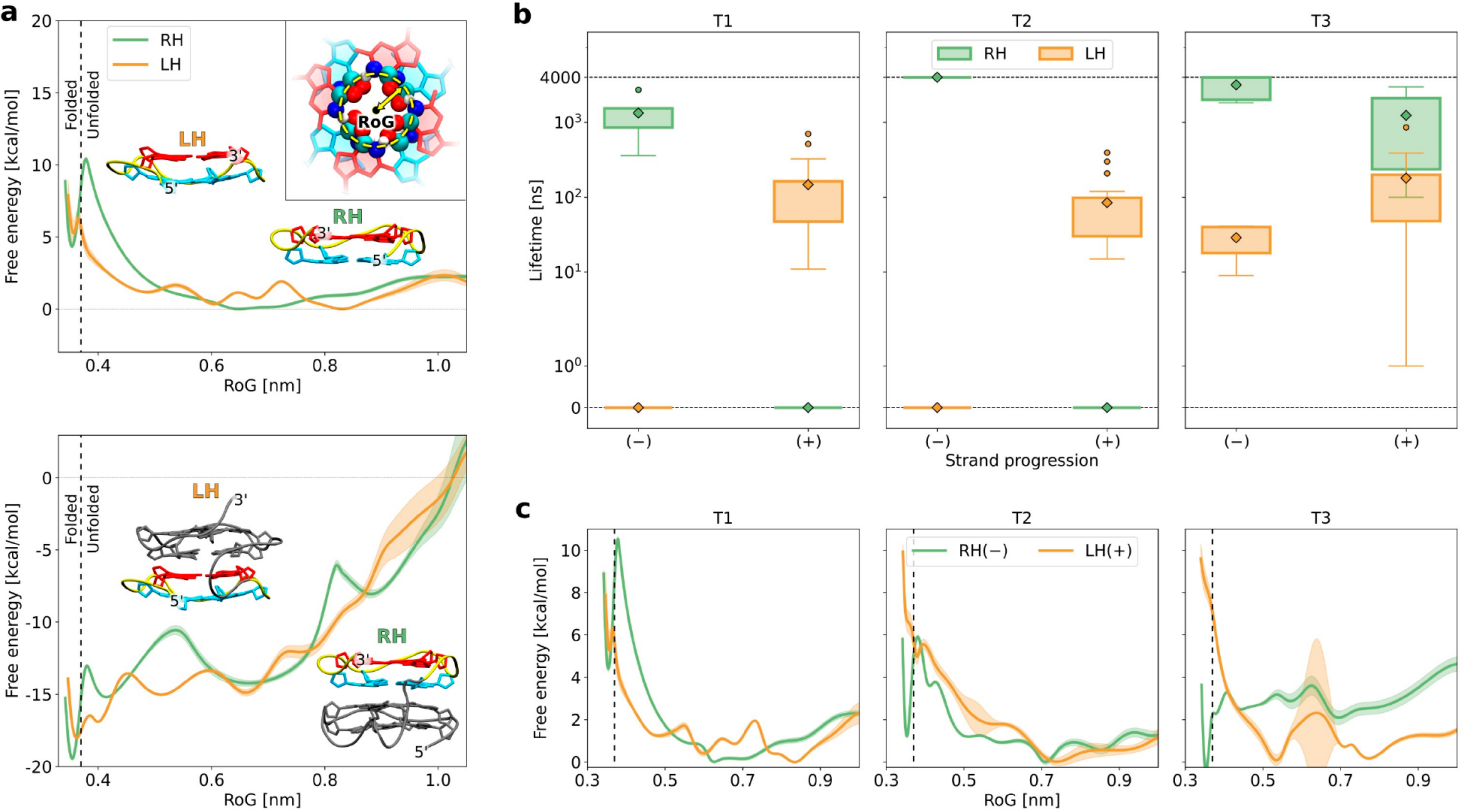
(a) Free energy profile for the folding of parallel G4s with RH (green) and LH (orange) helicity, in the absence (top) and presence (bottom) of an additional G4 block. The reaction coordinate is defined as the radius of gyration (RoG) of atoms forming the G4 central channel, as shown in the inset in the top right corner. The boundary separating the folded and unfolded states (RoG = 0.37 nm) is shown as a dashed vertical line. (b) Effect of the helicity (RH and LH), direction of strand progression [(+) and (−)], and loop length (T1, T2, and T3) on the lifetimes in the folded state of the parallel two-layer G4s. Diamonds represent the average lifetimes. (c) Free energy profiles for the folding of the RH(−) and LH(+) monomeric parallel G4s with T1, T2, and T3 loop lengths.

Specifically, to understand the observed dependence of G4 folding stability upon the helicity, we ran an exhaustive set of molecular dynamics (MD) simulations initiated at the RH and LH folded states of the T1 sequence and its variants with two or three thymines in each loop (T2 or T3, respectively). Additionally, for each folded state, we considered the two possible directions of the strand progression: clockwise (+) and anti-clockwise (−) (Figure S2 of the Supporting Information; see the Methods of the Supporting Information for details).

The average folded-state lifetimes (for lifetimes in individual MD runs, see Table S2 of the Supporting Information) extracted from these simulations (Figure 2b) reveal that the right-handed clockwise, RH(+), and left-handed anti-clockwise, LH(−), G4s undergo a very fast unfolding, especially evident for T1 and T2 loop variants (lifetimes of <1 ns). This might explain the absence of solved structures for these G4 types and strongly suggests that, as clarified below, in parallel G4s, for the RH fold to remain stable, it requires the (−) strand progression, while the LH fold necessitates the (+) strand progression.

Figure 2b further reveals that, among these energetically accessible folds, RH(−) has markedly longer lifetimes than LH(+), regardless of the loop length. Indeed, for T1, the lifetimes in the folded state are ∼1330 and 150 ns for RH(−) and LH(+), respectively, and this difference in kinetic stability becomes even more pronounced when the longer loops are present (T2/T3). This difference is also reflected by the folding free energy landscapes in Figure 2c, which generally show higher barriers to unfolding of the RH structures and, furthermore, predict their greater thermodynamic stability, as indicated by deeper folded state minima.

*Preference for the Right-Handed Helicity Largely Originates from the G-Tract Conformational Energetics*. Next, to examine if the observed differences in the stability between the RH(−) and LH(+) folds might arise from conformational preferences of the G-tracts, we calculated the free energy profile for the transition of an isolated G-tract (i.e., a guanine dinucleotide) between the RH and LH helicity (see the Supporting Information for details).

As seen in Figure 3a, the G-tract conformation present in the parallel RH G4s (RH *anti*/*anti*) is ∼3.5 kcal/mol more stable compared to that present in their LH counterparts (LH *anti*/*anti*). This finding indicates that the higher stability of RH G4s can indeed be largely attributed to the more energetically favorable conformation of the G-tracts themselves. To ensure the robustness of this crucial conclusion regarding the choice of force field, we recomputed the RH-to-LH transition free energy profile using the Amber OL15 force field.^37^ The resulting profile (Figure S3 of the Supporting Information) closely resembled the profile obtained with Amber bsc1 (Figure 3a), once again demonstrating a ∼4.0 kcal/mol preference for adopting the RH conformation.

**Figure 3:**
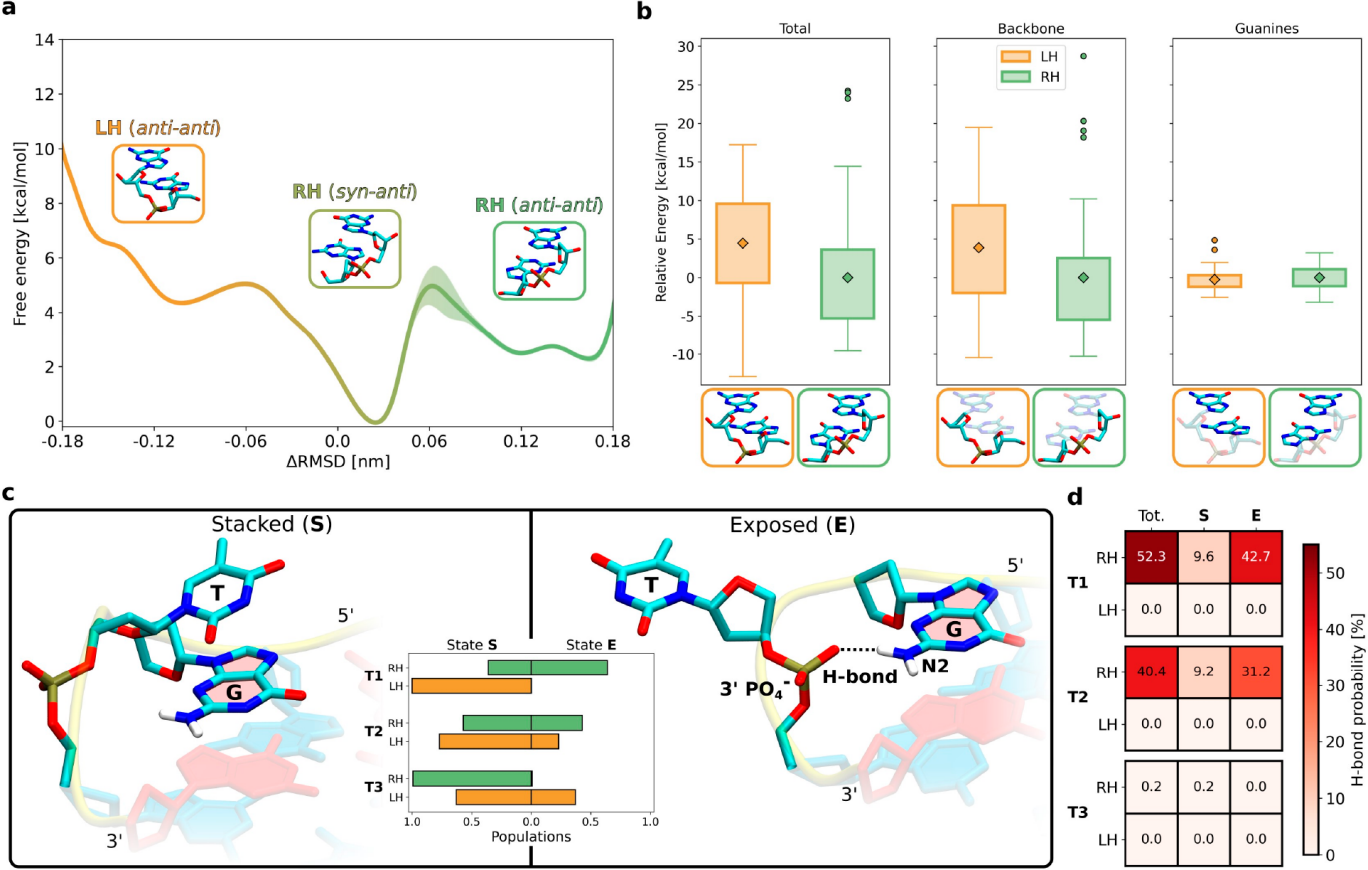
(a) Free energy profile for the transition of a guanine dinucleotide (mimicking a single G-tract) between LH and RH helicity. The reaction coordinate is defined as the difference in RMSD from the LH and RH G-tract conformations extracted from the full G4s. Insets link the values of the reaction coordinate to the conformations of the dinucleotide. (b) Comparison of density functional theory (DFT)-calculated energies for LH and RH conformations of guanine dinucleotides (G-tracts) sampled from all 10 models (i.e., 4 × 10 different conformations) representing the 2MS9 (LH) and 2N3M (RH) NMR G4 structures (for detailed energies, consult Table S3 of the Supporting Information). The total energies are decomposed approximately into the intrinsic energy of the sugar–phosphate backbone and the energy of the guanine–guanine interaction (for the MP2 results, see Figure S5 and Table S4 of the Supporting Information). (c) MD-derived populations of the “stacked” (S; left) and “exposed” (E; right) conformations for the RH and LH parallel G4s formed by the T1, T2, and T3 sequences (see also Figure S9 of the Supporting Information). All MD frames with the solvent-accessible surface area of 3′-thymine below 1.63 nm^2^ were assigned to the S conformation, and the remaining frames were assigned to the E conformation. (d) Probability of forming a specific hydrogen bond shown in panel c (right); Tot and S or E denote the total probability and its fractions corresponding to the S or E states, respectively.

Still, it was essential to investigate whether this preference could be influenced by a potential bias in empirical force fields toward right-handed helicity. Evaluation of the MD-generated ensemble of G-tract conformations against NMR data (Figure S4 of the Supporting Information) reveals a certain tendency for better representation of the RH conformation in MD simulations, regardless of the force fields applied, while maintaining an overall satisfactory agreement with an average root-mean-square deviation (RMSD) between NMR and MD structures below 0.074 nm. Therefore, to rule out the possibility of the observed higher stability of the RH conformation being a force field artifact, we computed the conformational energies of RH *anti*/*anti* and LH *anti*/*anti* G-tracts directly from the experimental G4 structures using a quantum chemical approach (see the Methods of the Supporting Information) and compared them in Figure 3b and Figure S5 of the Supporting Information. The resulting energy difference (Δ*E*) of ∼4.5 kcal/mol in favor of the RH conformation confirms the preference of the G-tract for the right-handed helicity at the quantum level. To obtain deeper insight into the origin of this preference, we decomposed Δ*E* into contributions as a result of the DNA backbone and the stacked guanine pair (Figure 3b). We found that the difference in the backbone energy (3.9 kcal/mol) is a primary contributor to overall Δ*E*, while the guanine–guanine interaction is nearly equivalent in both states (−0.2 kcal/mol). Consistent results were obtained for G-tracts derived from MD-generated ensembles, with an overall Δ*E* of 5.4 kcal/mol in favor of the RH conformation, predominantly arising from differences in backbone energy (see Figures S6 and S7 of the Supporting Information).

As an additional observation, our free energy profiles suggest that the RH *syn*/*anti* conformation (with a minimum at ∼0.025 nm) is considerably more stable than both *anti*/*anti* conformation (Figure 3a and Figure S3 of the Supporting Information). This somewhat unexpected result may be attributed to the tendency of the empirical force field to overly stabilize the *syn*/*anti* conformation, as evidenced by prior quantum chemical study.^38^

*Hydrogen-Bonding Properties Alone Cannot Account for the Stability Difference*. To investigate other possible factors contributing to the higher stability of RH G4s, we compared RH and LH folds in terms of intramolecular hydrogen bonds (Figure S8 of the Supporting Information).

The most prominent difference that we identified pertains to hydrogen bonds between the N2 amino groups in the 3′-terminal G-tetrad (red in Figure 1) and the phosphate group at the 3′ ends of each of the loops (Figure 3c). These specific hydrogen bonds are prevalent in RH G4s, while being almost completely lacking in LH G4s.

We further found that the formation of these hydrogen bonds requires 3′-phosphate in the loop to approach the guanine core, which is only possible when associated 3′-thymine is exposed to the solvent [the “exposed (E)” conformational state; see Figure 3c, right]. On the contrary, when 3′-thymine stacks on the 3′-terminal G-tetrad [the “stacked (S)” state; see Figure 3c, left], phosphate moves away from the guanine core and can no longer form a hydrogen bond to the N2 group. Accordingly, the inset in Figure 3c shows that the E state is highly populated (reaching up to 0.6) exclusively for the T1 and T2 variants of RH G4s, which are the only folds where the hydrogen bond forms with a significant probability (40–50%). State-wise decomposition of this probability in Figure 3d indeed demonstrates a strong correlation between the thymine conformation and hydrogen-bond formation, especially for the short-looped G4s. In contrast, LH G4s prefer to adopt the hydrogen-bond-incompetent S conformation, consistently with extensive thymine stacking revealed by the NMR solution structures of these folds.^39^

Interestingly, LH G4s demonstrate an alternative hydrogen-bonding pattern involving the O4 atom in deoxyribose (dRib; see Figure S8 of the Supporting Information). Specifically, for shorter loops (T1 and T2), hydrogen bonds form mostly between O4 and N2 in the 5′-G-tetrad (cyan in Figure 1), as previously confirmed by NMR studies,^25,26^ while for longer loops (T3), hydrogen bonds form mostly between O4 and N2 in the 3′-G-tetrad. Notably, despite being less stable overall, the LH folds show a higher propensity for intramolecular hydrogen bonds in the T3 case compared to their RH counterparts (Figure S8 of the Supporting Information). Thus, the differences in the hydrogen-bonding pattern appear to have a minimal impact on the G4 conformational equilibrium, especially when compared to the intrinsic preference of G-tracts to adopt the right-handed conformation, as mentioned above.

*Direction of Strand Progression Determines the Helicity of Parallel G4s in a Loop-Length-Dependent Manner*. As already indicated by the folded-state lifetimes (Figure 2b), in parallel G4s, RH helicity requires the (−) strand progression, while LH helicity is associated with (+) progression. The association was especially evident for shorter sequences (T1 and T2) and became weaker as the loop length increased (T3). To understand the structural underpinnings of this association and its dependence upon the loop length, we calculated the free energy profiles for the transition between LH and RH helicity, corresponding to the change in the average twist angle θ from −27° to +29° (see Figure S10 of the Supporting Information), separately for both possible directions of strand progression. To facilitate convergence, this was done for the DNA fragments consisting of two G-tracts connected by the thymine loop, where the reference RH and LH conformations were extracted from the full parallel G4s with either T1 or T3 loops (Figure 4a).

**Figure 4:**
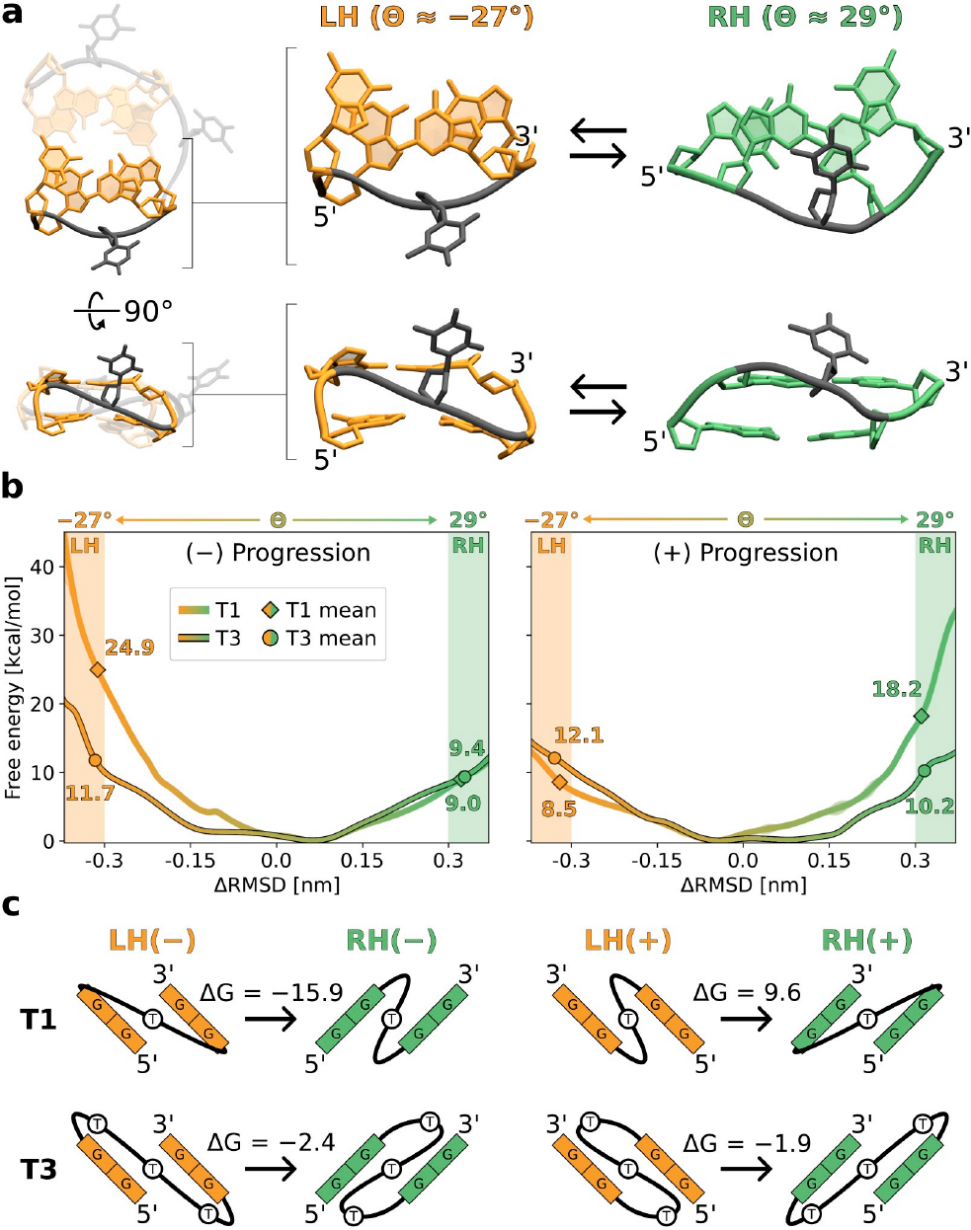
(a) Reaction coordinate used to investigate the relation between helicity and strand progression (ΔRMSD) was defined as the difference in RMSD from the reference LH and RH conformations of the indicated DNA fragment extracted from the appropriate full G4s. As shown in Figure S10 of the Supporting Information, the reference LH and RH conformations correspond to the average twist angle θ equal to −27° and 29°, respectively. For a depiction of all four cases considered, involving two possible strand progressions and two loop lengths (T1 and T3), consult Figure S11 of the Supporting Information. (b) Free energy profiles for the transition of the DNA fragment indicated in panel a between the LH (orange) and RH (green) conformations, computed separately for (+) and (−) strand progressions and two loop lengths (T1 and T3). Markers show the average free energy values characterizing the corresponding conformations. (c) Scheme explaining the dependence of helicity upon the direction of strand progression in parallel G4s (see the description in the text).

Consistent with the above observations, Figure 4b shows that (−) progression clearly favors the RH conformation (green), whose free energy, in the case of short T1 loops, is 15.9 kcal/mol lower than that of the LH conformation (in orange). Notably, as the loop length increases, this preference diminishes, resulting in a smaller free energy difference of 2.4 kcal/mol for T3. The opposite preference is found for (+) progression in the T1 case, where the LH state is 9.6 kcal/mol more stable than its RH counterpart (Figure 4b). However, when the loop is made longer (T3), the RH state again becomes slightly more favorable (by 1.9 kcal/mol), in agreement with the order of magnitude longer lifetime observed for the RH(+) folds of the T3 sequence (Figure 2b). The same general conclusions can be drawn from the free energy profiles describing the LH ⇌ RH transition in the complete G4 structures (Figure S12 of the Supporting Information).

The observed interdependence of the helicity and strand progression can be explained in simple geometric terms. As shown schematically in Figure 4c, the transition between the LH and RH conformations results in a significant change in the separation distance between the points where the loop attaches to the G-tracts. Specifically, in the case of (−) progression, to allow for LH helicity, the loops have to span a distance by 7 Å longer compared to the RH folds (Figure S13 of the Supporting Information). Conversely, for (+) progression, LH helicity requires a 6 Å shorter distance than RH (Figure S13 of the Supporting Information). Hence, in the LH(−) and RH(+) folds, the short T1 loops must adopt highly stretched configurations, making them energetically inaccessible, thereby leaving LH(+) and RH(−) as the only viable conformations. In contrast, the long T3 loops can span a longer distance between the attachment points without energetically unfavorable stretching, resulting in smaller free energy gaps and relatively low conformational preferences (Figure 4c). In the case of (+) progression, the minor destabilizing effect caused by the stretching of the T3 loop is actually outweighed by the more favorable conformation of G-tracts, which leads to a slight preference for RH helicity. Such a preference is also reflected by approximately 1 order of magnitude longer lifetimes of the RH(+) folds with T3 loops compared to their LH counterparts in Figure 2b. This finding suggests that RH helicity is more favorable irrespective of the strand progression direction when loops are at least 3 nucleotides long and, thus, places significant constraints on the sequences capable of forming left-handed G4s.

In summary, our MD-based free energy calculations and unbiased unfolding simulations clearly demonstrated that G4s with RH helicity are considerably more thermodynamically and kinetically stable than their LH counterparts. This prediction aligns with indirect experimental evidence, such as the scarcity of LH G4s and their sensitivity to sequence modifications.^26^

While the additional G4 block is necessary to stabilize the generally unstable two-layer G4s, the observed higher stability of RH structures persists regardless of the presence of this stabilizing block. Additionally, we found that RH and LH G4s require different directions of strand progression to be energetically accessible: RH adopts the anti-clockwise (−) direction, while LH adopts the clockwise (+) direction. In contrast, the RH(+) and LH(−) folds are strongly prohibited, particularly when short loops are present, which is consistent with the lack of determined G4 structures of these types.

To gain a molecular-level understanding of these findings, we thoroughly examined conformational transitions and fold-stabilizing interactions in a range of model systems and full G4s, utilizing free energy simulations complemented by quantum chemical calculations. Our results indicate that the higher stability of RH G4s is a result of a more favorable conformation of the sugar–phosphate backbone in their G-tracts, with the guanine–guanine stacking interaction being very similar between the RH and LH structures. It might be anticipated that the observed preference for the RH backbone conformation would be even more pronounced in longer G-tracts, potentially explaining the lack of solved LH G4s with three or more guanine layers.

Interestingly, the hydrogen-bonding pattern, while different between RH and LH G4s, does not appear to contribute markedly to the stability difference.

Finally, we also offered a simple explanation of how the helicity and direction of strand progression in parallel G4s are interconnected in a loop-length-dependent manner. Essentially, accommodating short 1 or 2 nucleotide loops between the successive G-tracts requires the loop attachment points to be in close proximity. This geometric criterion is met only by the RH(−) and LH(+) folds, making them energetically accessible. In contrast, when loops are 3 nucleotides in length or longer, the association between helicity and strand progression becomes less strict, favoring RH helicity regardless of the direction of strand progression. This observation suggests the following design principle: achieving LH helicity in G4s demands enforcing a clockwise (+) strand progression (e.g., by an additional G4 block) and utilizing sequences with loops not exceeding 2 nucleotides in length.

## Data Availability

Data associated with this article are available under this link: 10.34808/w2df-6642.

## Supporting Information Available

Methods, Tables S1–S10, and Figures S1–S15 (PDF)
Steered-MD-based folding simulations for RH(−) and LH(+) structures with T1 loops (Movie S1) (MP4)
