# Supplementary material for: Why Are Left-Handed G-Quadruplexes Scarce?
Source: J Phys Chem Lett. 2024 Mar 13;15(11):3142–8. doi: 10.1021/acs.jpclett.3c03589 (PMC10961827; doi:10.1021/acs.jpclett.3c03589)
Supplement: Supplementary file 1 — jz3c03589_si_001.pdf [file jz3c03589_si_001.pdf]

Supporting Information for: Why Are Left-Handed G-Quadruplexes Scarce?

Michał Jurkowski,[†] Mateusz Kogut,[†] Subrahmanyam Sappati,[†,‡] and Jacek Czub[*,†,‡]

†*Department of Physical Chemistry, Gdańsk University of Technology, Narutowicza St 11/12, 80-233 Gdańsk, Poland*

‡*BioTechMed Center, Gdańsk University of Technology, Narutowicza St 11/12, 80-233, Gdańsk, Poland*

E-mail: jacek.czub@pg.edu.pl

SI Methods

Simulation systems

The simulated G4 systems contained unimolecular two-layered G-quadruplexes with all-parallel topology, in either monomeric or dimeric form whose initial geometries were taken from NMR solution structures or obtained by *in silico* building (see "Building procedure" section for details). In all systems, the solutes were situated within a dodecahedral box comprised of TIP3P water molecules,[1] ensuring a minimum distance of 1.2 nm between any solute atom and the box's edges. In each case, we added the appropriate number of K^+ and Cl^- ions to neutralize the system and maintain a physiological ionic strength of 150 mM. Additionally, K^+ ions were introduced into the central channel of G4. For all MD simulations we used AMBER bsc1 force field[2] (unless stated otherwise; see "Free energy calculations"

subsection) with ions' parameters corrections.[3]

To calculate the free energy profiles for the folding of G4s with right- and left-handed helicity (Fig. 2a), we prepared the following systems:

[A.1] G4 dimer composed of two left-handed, two-layered blocks stacked together, and connected by a TT linker (see LH inset in the bottom plot of Fig. 2a). The sequences of the blocks were $(TGG)_4$ and $G(TGG)_3TGTT$. The initial geometry was taken from the NMR structure (2MS9).[4]

[A.2] G4 dimer of right-handed two-layered blocks stacked together and connected by a single-thymine linker (see RH inset in bottom plot of Fig. 2a). The sequences of the blocks were $(TGG)_3(TTG)_2$ and $(GGT)_4$, and the initial geometry was taken from the NMR structure (2N3M).[5]

[A.3] Left-handed G4 monomer with $GG(TGG)_3$ sequenence and initial geometry extracted from the 2MS9 structure (see LH inset in the top plot of Fig. 2a).

[A.4] Right-handed G4 monomer with $GG(TGG)_3$ sequenence and initial geometry extracted from the 2N3M structure (see RH inset in the top plot of Fig. 2a.).

To determine the effect of helicity (RH, LH), the direction of strand progression [(+), (−)], and loop length (T1, T2 and T3) on the unfolding times of the all-parallel two-layered G-quadruplexes (Fig. 2b), the following G4 monomers with the sequence $GGT_nGGT_nGGT_nGG$ were prepared by *in silico* building procedure (described below):

[B.1] LH G4 with clockwise (+) strand progression and three different loop lengths, $n = 1, 2$ or 3 (referred to as T1, T2, T3) (see LH(+) in Fig. S2).

[B.2] LH G4 with anticlockwise (−) strand progression and three different loop lengths (T1, T2, T3) (see LH(−) in Fig. S2).

[B.3] RH G4 with (+) strand progression and three different loop lengths (T1, T2, T3) (see RH(+) in Fig. S2). via

[B.4] RH G4 with (−) strand progression and three different loop lengths (T1, T2, T3) (see RH(−) in Fig. S2).

Initial geometries for all systems [B.1]–[B.4] and merged trajectories representing folded state ensembles for energetically accessible structures (see Results) can be found under this link: https://doi.org/10.34808/w2df-6642.

To study the energetics of the conformational transition of a G-tract between the LH and RH helicity (Fig. 3a), we prepared the following system:

[C.1] A guanine dinucleotide (GpG) with the initial configuration extracted from the 5'-end of the block formed by $(TGG)_4$ sequence from 2MS9 NMR structure. 5'- and 3'-hydroxyl groups were capped by hydrogens (see inset in Fig. 3a).

To study the relation between helicity and strand progression in all-parallel G-quadruplexes (Fig. 4), we prepared the following systems:

[D.1] Two DNA oligonucleotides with the sequences GGTGG and GGT_3GG corresponding to two G-tracts connected by either one- or three-thymine loop with (+) strand progression. Initial configurations were extracted from the 5'-end of the [B.1] structure with T1 or T3 loops. The 3'-end oxygen (O3') was capped with a hydrogen atom.

[D.2] Two DNA oligonucleotides with the sequences GGTGG and GGT_3GG corresponding to two G-tracts connected by either one- or three-thymine loop with (−) strand progression. Initial configurations were extracted from the 5'-end of the [B.4] structure with T1 or T3 loops. The 3'-end oxygen (O3') was capped with a hydrogen atom.

MD protocol

The MD simulations were performed using Gromacs 2020[6] with Plumed 2.6 plugin.[7] Simulations were conducted in the NPT ensemble, with the temperature kept at 300 K and using the v-rescale thermostat[8] and the pressure kept at 1 bar using Parrinello-Rahman barostat.[9] Periodic boundary conditions were applied in 3D, and electrostatic interactions were calculated using the Particle Mesh Ewald (PME)[10] method with a real-space cutoff of 1.2 nm and a Fourier grid spacing of 0.12 nm. A cut-off of 1.2 nm was used for Lennard-Jones potential representing van der Waals interactions. All bond lengths were constrained using

P-LINCS[11] for DNA and SETTLE[12] for water. The equations of motion were integrated using the leap-frog algorithm with a 2 fs time step.

Lifetime estimation

To estimate the folded-state lifetimes (unfolding times), of parallel G4s with varying helicities, strand progression directions, and loop lengths, we conducted a comprehensive set of unbiased MD simulations. These simulations were initiated from the folded state for systems [B.1]–[B.4], each featuring three different loop lengths: T1, T2, and T3. For each of these 12 systems, we carried out 5 independent simulation replicas, each with a maximum runtime of 4 μs. These simulations were terminated upon the unfolding of the G-quadruplex. We considered the G-quadruplexes to be in an unfolded state when the radius of gyration (RoG) of the atoms forming the G4 central channel (as shown in the inset in Fig. 2a) exceeded 0.37 nm, which corresponds to the transition state maximum in the folding free energy profiles (Fig. 2c).

Some of the energetically accessible folds which were to be further analyzed, particularly LH(+) with all loop length variants, exhibited relatively short lifetimes, which resulted in short total sampling time of their folded state. Hence, for these systems we simulated additional replicas to generate the folded state ensembles of at least 1.5 μs-length. Total sampling times for all systems can be found in Table S1.

Free energy calculations

Folding free energy profiles

To investigate the effect of the helicity of G-quadruplexes on their stability in the folded state, we calculated the free energy profiles for the folding of a set of dimeric (systems [A.1] and [A.2]) and monomeric two-layer parallel G-quadruplexes (systems [B.1] and [B.4]). To this end, we used replica-exchange umbrella sampling[13] (REUS) along the reaction coordinate

defined as the radius of gyration (RoG) of the guanine atoms forming the G4 central channel (O6, C6, N1 and H1; see inset in Fig. 2a). In each case, we sampled the RoG coordinate in the range from 0.35 nm, corresponding to the the fully folded G4, to 1.4 nm, corresponding to the fully-unfolded G4. Table S7 provides comprehensive details on the number of REUS windows, spring constants, and the positions of biasing potential used for specific simulations. The initial configurations for the REUS windows were generated through additional unfolding simulations, where we enforced a RoG coordinate change from 0.35 to 1.4 nm at a speed of 0.004 nm/ns, using a harmonic moving potential with a spring constant of 10 kJ/(mol·nm^2). All windows were simulated for 1.5 μs and first 0.5 μs were discarded from analysis as equilibration.

The free energy profiles were determined using the weighted histogram analysis method (WHAM).[14] Uncertainties in the free energy were estimated using a bootstrap approach taking into account autocorrelation of the time series of the reaction coordinate in each window. The same procedure was applied to all free energy calculations described below.

Free energy profile for the transition between the RH and LH helicity

To calculate the free energy profiles for the conformational transition of a fully-solvated GpG dinucleotide (representing a single G-tract; system [C.1]), between LH and RH helicity states, we used replica-exchange umbrella sampling (REUS). REUS simulations were performed utilizing two different force fields: Amber bsc1[2] and Amber OL15.[15] The reaction coordinate was defined as the difference between the heavy-atom root mean square deviation (RMSD) from the reference left- and right-handed conformations of a two-guanine G-tract ($\Delta RMSD$). The reference LH and RH conformations were extracted from the NMR structures of all-parallel two-layered left- and right-handed G-qudruplexes, respectively (2MS9 and 2N3M).[4,5] As the conformation of the terminal atoms (O5' C5' and O3') does not affect the G-tract helicity, they were omitted from the definition of the reaction coordinate.

We utilized 10 REUS windows to cover the entire range of the reaction coordinate, span-

ning from -0.18 to 0.18 nm. With our setup, these endpoints correspond to the LH and RH conformations, respectively. Within each of these windows, we applied a harmonic potential to restrict the system's motion along the reaction coordinate, and the specific center positions and spring constants can be found in Table S8. Additionally, to ensure a unique correspondence between the endpoints of the reaction coordinate and the reference conformations, we applied one-sided harmonic potentials that penalize RMSD values exceeding 0.22 nm with respect to individual references. To obtain initial configurations of the guanine dinucleotide for the REUS windows, we performed an additional steered MD simulation. In this simulation, we enforced the transition from the LH to RH conformation by minimizing the RMSD from the RH reference structure, using a harmonic potential with a force constant of 10 kJ/(mol·nm^2) and a motion rate of 0.0012 nm/ns. All windows were simulated for 1 μs, with exchanges between neighboring replicas attempted every 2 ps.

Relationship between G4 helicity and direction of strand progression.

To explain the association between helicity and direction of strand progression in parallel G4s, we used REUS method to determine a set of free energy landscapes governing the transition between the LH and RH conformation for DNA oligonucleotides comprising of two G-tracts connected by one- or three-thymine loops (GGTGG and GGTTTGG, respectively). This was done separately for the (+) and ($-$) strand progression (the systems [D.1] and [D.2], respectively; see Fig. S11). The reaction coordinate was defined as the difference between the heavy-atom RMSD from the reference LH and RH conformations of a considered oligonucleotide ($\Delta RMSD$). The reference LH(+) and RH(+) conformations were extracted from the equilibrated G-quadruplexes in the systems [B.1] and [B.3], respectively, while LH($-$) and RH($-$) from the equilibrated G-quadruplexes in the systems [B.2] and [B.4], respectively.

We used 16 REUS windows to span the entire reaction coordinate, ranging from -0.40 to 0.40 nm, with these endpoints representing the LH and RH conformations of the oligonu-

cleotides in our setup. Within each of these windows, we applied a harmonic potential to restrict the system's motion along the reaction coordinate, and the specific center positions and spring constants can be found in Table S9. To ensure a thorough sampling of the energetically unfavorable LH(−) and RH(+) conformations, within the region of the reaction coordinate corresponding to these conformations, we utilized a steeper biasing potential with higher force constants. Additionally, to guarantee a unique correspondence between the endpoints of the reaction coordinate and the reference conformations, we applied one-sided harmonic potentials that penalize RMSD values exceeding 0.48 nm with respect to individual references. To generate initial configurations for the REUS windows, we performed steered MD simulations in which the transition of the oligonucleotides between the reference LH and RH conformations was enforced by a harmonic potential acting along the RMSD from the respective conformations and moving at the rate of 0.002 nm/ns. Concomitantly, a spring constant was also gradually increased from 10 to 80 kJ/(mol·nm^2). All windows were simulated for 1 μs, with exchanges between neighboring replicas attempted every 2 ps.

Helicity change in a complete G-quadruplex

To calculate the free energy profile for the transition between LH and RH helicity of the complete G4 structures with (−) and (+) strand progression and two different loop lengths (T1 and T3), we used replica exchange umbrella sampling (REUS). The reaction coordinate was defined as the difference between the heavy-atom root mean square deviation (RMSD) from the reference left- and right-handed conformations of the guanine core ($\Delta RMSD$). The reference LH(+) and RH(+) conformations were extracted from the equilibrated G-quadruplexes in the systems [B.1] and [B.3], respectively, while LH(−) and RH(−) from the equilibrated G-quadruplexes in the systems [B.2] and [B.4], respectively.

We used 17 REUS windows to cover the space of the reaction coordinate ranging from −0.28 nm to 0.28 nm which corresponds to LH and RH conformations respectively. Within each of these windows, we applied a harmonic potential to restrict the system's motion

along the reaction coordinate, and the specific center positions and spring constants can be found in Table S10. To provide a sufficient sampling of entire range of the reaction coordinate, especially regions corresponding to LH(−) and RH(+) conformations characterized by steep free energy slope, we adjusted the potential spring constants in appropriate windows. Additionally, to guarantee a unique correspondence between the endpoints of the reaction coordinate and the reference conformations, we applied one-sided harmonic potentials that penalize RMSD values exceeding 0.36 nm with respect to individual references. To generate initial configurations for the REUS windows, we performed steered MD simulations in which the transition of the oligonucleotides between the reference LH and RH conformations was enforced by a harmonic potential moving along the RMSD from the respective conformation with rate of 0.001 nm/ns. Spring constant of this potential also gradually increased from 10 to 100 kJ/(mol·nm^2). All windows were simulated for 0.5 μs, with exchanges between neighboring replicas attempted every 2 ps. For the purpose of free energy visualization, the unbiased ensemble determined via WHAM was projected on the more natural coordinate, i.e., the G4 twist angle Θ defined in Fig S10.

Quantum chemical calculations

To compare the conformational energies of the RH and LH states of the two-guanine G-tract (i.e., a guanine dinucleotide; see Fig. 3b) at the quantum-mechanical level, we used both DFT and wave function-based methods implemented in Orca 5.0.[16] Specifically, we performed single-point energy calculations for 40 left-handed and 40 right-handed conformations of G-tracts extracted from all 10 models (4 G-tracts×10 models) of 5'-end block of 2MS9 (LH) and 3'-end block of 2N3M (RH) NMR G4 structures. Similar calculations were performed for 30 left-handed and 30 right-handed conformations of the 5'-terminal G-tract randomly selected from the MD-generated conformational ensembles of complete two-layer parallel G4s in LH or RH states (systems [B.1] and [B.4], respectively). 5' and/or 3'-ends of the extracted GpG dinucleotides were capped with hydrogen atoms. For the DFT calculations, we used B3LYP

exchange-correlation functional[17] with the D3 dispersion correction[18] and the 6-311G(d) basis set.[19] In the wave-function approach, we used the spin-component-scaled second-order Møller–Plesset perturbation theory (SCS-MP2)[20] with the cc-pVTZ basis sets.[21] The same two techniques were used to calculate conformational energies of the backbone and guanine components of the dinucleotides. The geometries of these two components were extracted from the originally selected sets of guanine dinucleotide conformations and capped with hydrogen atoms at C1' and N9 sites, respectively.

Building procedure

For systems [B.1] – [B.4], we prepared the initial structures of the G-quadruplexes using our developed *de novo* building procedure. This involved guiding an unfolded DNA oligonucleotide with the sequence $GGT_nGGT_nGGT_nGG$ ($n = 1, 2$ or 3) to fold into the desired G4 structure.

Essentially, we gradually threaded the oligonucleotide onto a reference structure of the guanine core in a G-tract by G-tract manner, starting from the 5'-end. This process utilized steered molecular dynamics and a set of four reference structures, with each one involving one more G-tract than the previous one (Fig. S14). For the LH G4s in the systems [B.1] and [B.2] the reference structures were taken from the NMR structure of the left-handed $(GGT)_4$ block (PDB id: 2MS9). Similarly, for the RH G4s in the systems [B.3] and [B.4] the reference structures were taken from the NMR structure of the right-handed $(TGG)_4$ block (PDB id: 2M3N). In four steps of the procedure, a moving harmonic potential with a spring constant of 10000 kJ/(mol·nm^2) was applied to RMSD from the reference structure to drive the consecutive G-tracts to fold into the guanine core with a desired helicity and strand progression (over 12 ns per step; see Movie S1). To expedite calculations, this was carried out in implicit solvent conditions with the dielectric constant set to 15. This value was fine-tuned for our specific procedure. It is low enough to prevent DNA strand collapse due to weak phosphate-phosphate repulsion observed at high dielectric constants, yet high enough

to mitigate strong phosphate-phosphate repulsion favoring extended DNA conformations over G-quadruplexes at low dielectric constants.

After the complete guanine core formation, the newly folded G4 structures were subjected to a 12 ns simulation in the same medium to facilitate local relaxation of the twist angle between two G-tetrads. This relaxation was achieved by restraining both G-tetrads separately, ensuring that the RMSD from the reference G-tetrad structure remained below 0.05 nm. A harmonic potential with a spring constant of 5000 kJ/(mol·nm^2) was used for this purpose.

Further relaxation of the obtained structures was carried out in explicit solvent conditions described in the "Simulation systems" section. To this end, the entire system was first gradually heated from 300 K to 400 K for 20 ns and then, for the next 20 ns, cooled back to 300 K. During the cooling process the restraints imposed on the guanine tetrads were progressively removed.

All systems prepared using the procedure described above are available under this link: https://doi.org/10.34808/w2df-6642.

To validate our *de novo* building method, we compared the folded-state lifetimes of our generated G4 structures with those calculated for the corresponding G4s determined by NMR spectroscopy. Specifically, we focused on LH(+) and RH(−) G4s (systems [B.1] and [B.4], respectively), each having experimentally-solved counterparts (systems [A.3] and [A.4], respectively). The resulting distribution of the folded-state lifetimes (Fig. S15) displayed remarkable similarity, confirming the effectiveness of our procedure in generating well-structured and properly relaxed G-quadruplexes.

Table S1: Summary of PDB codes for high-resolution structures of left-handed G-quadruplexes, featuring sequences composing the two G4 blocks within each structure. The connectivity status of G4 blocks, whether connected by a linker (T or TT) or disconnected (-), is highlighted.

PDB code	5'-end block sequence	linker	3'-end block sequence
2MS9[4]	TGGTGGTGGTGG	TT	GTGGTGGTGGTGTT
4U5M[4]	TGGTGGTGGTGG	TT	GTGGTGGTGGTGTT
6GZ6[22]	GTGGTGGTGGTG	TT	GTGGTGGTGGTGT
6FQ2[22]	GTGGTGGTGGTG	-	GTGGTGGTGGTG
6JCE[23]	GGTTGGTGTGG	TT	GGTTGTGGTGGTGGTG
6QJO[23]	GGTTGGTGTGG	TT	GGTTGTGGTGGTGGTG
7D5D[24]	GGTGTGTGGTGG	T	GTGGTGGTGGTGTT
7D5E[24]	GGTGTGTGTGTGG	T	GTGGTGGTGGTGTT
7D5F[24]	GGTGTGTGTGTGTG	T	GTGGTGGTGGTG
7DFY[25]	GGTGGTGGTGTG	TT	GGTGGTGGTGTG

Table S2: Folded-state lifetimes (in ns) as observed in individual MD simulations of all parallel G-quadruplexes considered in this work. LH and RH stand for left- and right-handed helicity, respectively; (+) and (−) denote clockwise and anticlockwise strand progression, respectively; T1–T3 stand for three different loop lengths. Individual runs in which G4s' folded state lifetimes exceeded 4000 ns are denoted as ”>4000.0” while in those where G4s unfolded immediately at the beginning of a production run as ”<1.0”.

	T1				T2				T3			
	(−)		(+)		(−)		(+)		(−)		(+)	
	LH	RH	LH	RH	LH	RH	LH	RH	LH	RH	LH	RH
1	<1.0	853.0	122.0	<1.0	<1.0	>4000.0	73.0	<1.0	9.0	>4000.0	46.0	100.0
2	<1.0	360.0	177.0	<1.0	<1.0	>4000.0	15.0	<1.0	36.0	>4000.0	86.0	2097.0
3	<1.0	1535.0	20.0	<1.0	<1.0	>4000.0	31.0	<1.0	41.0	>4000.0	150.0	758.0
4	<1.0	2722.0	11.0	<1.0	<1.0	>4000.0	39.0	<1.0	40.0	1828.0	199.0	2978.0
5	<1.0	1184.0	98.0	<1.0	<1.0	>4000.0	38.0	<1.0	18.0	2001.0	190.0	236.0
6	-	-	48.0	-	-	-	36.0	-	-	-	857.0	-
7	-	-	515.0	-	-	-	29.0	-	-	-	105.0	-
8	-	-	65.0	-	-	-	103.0	-	-	-	75.0	-
9	-	-	73.0	-	-	-	300.0	-	-	-	10.0	-
10	-	-	13.0	-	-	-	43.0	-	-	-	326.0	-
11	-	-	30.0	-	-	-	98.0	-	-	-	8.0	-
12	-	-	182.0	-	-	-	42.0	-	-	-	122.0	-
13	-	-	51.0	-	-	-	30.0	-	-	-	1.0	-
14	-	-	91.0	-	-	-	31.0	-	-	-	54.0	-
15	-	-	702.0	-	-	-	208.0	-	-	-	371.0	-
16	-	-	47.0	-	-	-	68.0	-	-	-	199.0	-
17	-	-	325.0	-	-	-	27.0	-	-	-	389.0	-
18	-	-	77.0	-	-	-	119.0	-	-	-	38.0	-
19	-	-	-	-	-	-	30.0	-	-	-	-	-
20	-	-	-	-	-	-	393.0	-	-	-	-	-
21	-	-	-	-	-	-	23.0	-	-	-	-	-

Table S3: Relative DFT energies (in kcal/mol) for 40 G-tract conformations extracted from the 2MS9 (LH) and 2N3M (RH) NMR G-quadruplex structures. Total conformational energy (Total) is approximately decomoposed into the intrinsic backbone energy and guanine-guanine interaction energy (Backbone and Guanines). Relative energies were calculated as the difference between the energy of a given configuration and the average energy in the RH state.

	Total		Backbone		Guanines	
	LH	RH	LH	RH	LH	RH
1	-3.32	6.09	-7.62	5.51	-1.01	-0.42
2	5.49	23.21	2.30	19.04	-0.89	2.06
3	3.22	-2.46	-2.96	-2.97	1.47	0.31
4	16.90	-1.15	11.23	-4.75	0.50	2.31
5	11.19	-6.07	7.04	-9.15	-0.26	2.32
6	3.22	-3.34	-1.19	-4.83	-0.07	-0.18
7	10.79	-5.11	5.38	-6.30	0.78	0.03
8	-1.21	-4.26	-8.77	-6.17	3.62	1.90
9	10.31	-2.42	2.66	-7.39	1.87	3.21
10	-2.27	-3.74	-7.14	-2.59	0.89	-1.88
11	9.32	-8.00	18.34	-6.66	-2.56	-0.81
12	6.04	-1.28	3.83	-1.05	-0.27	-1.59
13	4.57	-5.75	1.87	-3.90	-0.94	-1.64
14	-9.27	-5.01	-9.36	-3.76	-1.52	-0.06
15	1.33	-9.14	2.19	-8.39	-1.62	-0.26
16	12.07	-4.55	12.90	-5.18	-0.43	-0.40
17	1.69	2.05	3.31	1.53	-1.31	0.90
18	-3.97	0.66	-3.27	-0.40	-1.15	1.37
19	-1.72	-2.49	-3.76	-1.12	-0.69	-0.64
20	13.85	14.42	15.69	18.20	-1.12	1.39
21	0.60	6.90	2.51	9.62	-1.89	-1.62
22	-3.23	9.81	-2.39	9.82	-2.47	-1.13
23	2.30	-5.86	1.01	-3.61	-0.40	-1.37
24	-3.13	3.75	1.00	3.31	-0.59	-0.76
25	9.22	-5.87	14.08	-3.36	-2.33	-1.09
26	14.49	-7.67	6.13	-6.96	4.85	-0.03
27	-12.91	-4.84	-10.45	-2.66	-2.11	-1.21
28	-0.52	-3.74	-1.82	-3.07	0.11	-0.23
29	-4.18	5.87	-3.98	8.43	-1.73	-3.17
30	8.71	-9.55	10.42	-6.29	-0.24	-0.91
31	6.08	-6.20	7.01	-5.23	-0.62	-0.71
32	15.62	4.58	13.97	10.22	0.24	-1.77
33	17.24	-3.43	19.48	-4.98	-0.38	1.42
34	2.34	24.21	4.60	28.72	-0.13	-1.29
35	1.63	-1.86	2.66	-5.04	1.97	2.36
36	3.49	-3.40	5.19	-4.40	0.78	-0.21
37	7.02	-8.80	8.89	-10.27	-0.10	0.87
38	8.48	24.00	11.65	20.32	-0.07	-0.18
39	12.00	-3.21	13.47	-6.53	1.33	2.09
40	5.27	3.63	9.03	2.31	-1.22	1.01

Table S4: Relative SCS-MP2 energies (in kcal/mol) for 40 G-tract conformations extracted from the 2MS9 (LH) and 2N3M (RH) NMR G-quadruplex structures. Total conformational energy (Total) is approximately decomposed into the intrinsic backbone energy and guanine-guanine interaction energy (Backbone and Guanines). Relative energies were calculated as the difference between the energy of a given configuration and the average energy in the RH state.

	Total		Backbone		Guanines	
	LH	RH	LH	RH	LH	RH
1	-3.40	6.20	-9.14	6.16	0.32	-0.78
2	3.62	19.50	0.10	17.33	0.17	0.50
3	2.16	-2.51	-3.78	-2.96	1.40	0.10
4	14.62	-3.06	9.16	-4.90	0.64	0.57
5	9.63	-7.88	5.22	-9.26	0.36	0.34
6	1.71	-2.88	-3.24	-4.74	0.77	0.07
7	9.90	-5.48	4.30	-6.45	1.28	-0.48
8	-4.93	-6.41	-10.20	-6.08	1.95	-0.14
9	7.05	-4.93	1.03	-7.55	1.25	0.82
10	-3.61	-1.44	-8.06	-2.32	0.91	-0.06
11	11.87	-7.36	19.15	-6.60	-1.00	-0.33
12	5.40	0.46	1.52	-0.95	1.23	-0.43
13	2.99	-4.86	-0.42	-3.08	0.04	-1.94
14	-9.25	-5.53	-11.22	-3.79	-0.29	-0.53
15	2.40	-9.52	0.66	-8.30	0.64	-1.16
16	11.33	-5.44	12.29	-5.39	-0.91	-0.52
17	0.50	2.07	0.43	2.12	-0.02	0.21
18	-3.20	-0.79	-4.92	0.07	0.92	-0.20
19	-2.40	-1.83	-5.34	-1.70	-0.56	0.54
20	12.63	14.42	14.90	18.63	-1.02	1.01
21	1.64	8.72	1.85	9.64	0.63	-0.21
22	-2.55	11.92	-3.40	9.79	-0.85	0.38
23	2.23	-5.52	0.63	-4.05	0.30	-0.84
24	-2.82	6.08	1.15	4.04	-0.57	0.81
25	11.94	-4.42	15.14	-3.34	-0.45	0.12
26	11.06	-6.94	6.57	-7.22	3.11	0.31
27	-12.84	-3.56	-12.11	-2.83	-0.51	0.08
28	-0.56	-2.60	-1.48	-2.67	0.38	0.14
29	-4.80	8.32	-5.13	7.82	-0.75	-0.27
30	4.91	-9.36	8.26	-7.35	-1.83	-0.51
31	7.14	-6.76	7.45	-5.39	0.58	-0.74
32	12.98	5.21	12.79	9.66	-0.68	-0.84
33	15.02	-4.71	18.61	-5.02	-1.27	0.17
34	-0.19	25.34	3.56	29.75	-1.53	-0.42
35	-0.82	-2.79	2.08	-4.83	0.16	1.17
36	1.54	-3.08	4.05	-4.14	-0.09	0.06
37	7.97	-8.81	9.48	-10.03	0.65	0.58
38	7.31	21.12	10.82	19.46	-0.40	0.41
39	9.42	-4.04	12.58	-6.24	-0.32	1.02
40	3.64	3.15	8.24	2.73	-1.98	0.98

Table S5: Relative DFT energies (in kcal/mol) for 30 guanine dinucleotides (G-tracts) configurations in both LH and RH conformation randomly selected from our MD-generated ensembles (obtained with the Amber bsc1 force field). Total conformational energy (Total) is approximately decomposed into the intrinsic backbone energy and guanine-guanine interaction energy (Backbone and Guanines). Relative energies were calculated as the difference between the energy of a given configuration and the average energy in the RH state.

	Total		Backbone		Guanines	
	LH	RH	LH	RH	LH	RH
1	-2.76	-1.69	2.04	-1.31	0.08	1.55
2	11.77	-7.70	10.48	-4.25	3.61	-3.26
3	11.85	3.69	14.74	2.08	2.18	2.44
4	0.23	4.55	2.63	0.83	2.91	3.65
5	3.78	14.77	5.03	13.11	1.64	3.45
6	18.01	4.56	17.74	2.87	6.55	-0.78
7	5.30	-0.53	2.36	-2.12	6.69	1.11
8	1.92	-1.86	6.52	-3.16	-0.20	0.46
9	-3.96	2.80	3.60	-1.01	-4.15	3.19
10	7.99	-6.28	1.60	-2.01	9.23	-2.30
11	5.95	0.08	6.35	2.33	-2.57	-1.47
12	3.93	-6.21	7.99	-5.92	-3.22	-1.52
13	2.24	-2.38	5.85	1.32	1.52	-3.09
14	4.65	-3.68	4.76	-1.70	1.89	-4.40
15	-1.52	2.31	2.09	-3.61	1.78	5.54
16	3.88	24.96	9.54	12.59	-3.14	11.88
17	13.71	0.94	18.33	-4.35	-2.53	8.38
18	10.15	6.44	8.36	2.20	4.45	3.47
19	20.43	-0.22	13.45	-1.84	8.84	2.96
20	13.31	-1.73	6.44	0.43	5.83	0.26
21	7.91	-4.33	14.00	4.23	-4.34	-6.18
22	9.03	7.55	11.24	4.12	-1.25	-0.69
23	3.71	-0.87	9.46	3.16	-2.78	-3.94
24	7.07	2.22	8.13	1.05	0.78	0.35
25	4.67	-11.19	10.67	-8.19	-4.08	-2.52
26	3.33	-11.50	5.82	-10.43	-0.01	-3.40
27	-1.62	-4.29	1.33	-2.34	-3.19	-0.63
28	1.66	-1.31	7.11	-1.73	-3.30	-1.04
29	-7.56	3.06	0.14	5.96	-3.55	-4.21
30	3.76	-12.14	-0.49	-2.33	4.12	-9.26

Table S6: Relative SCS-MP2 energies (in kcal/mol) for 30 guanine dinucleotides (G-tracts) configurations in both LH and RH conformation randomly selected from our MD-generated ensembles (obrained with the Amber bsc1 force field). Total conformational energy (Total) is approximately decomposed into the intrinsic backbone energy and guanine-guanine interaction energy (Backbone and Guanines). Relative energies were calculated as the difference between the energy of a given configuration and the average energy in the RH state.

	Total		Backbone		Guanines	
	LH	RH	LH	RH	LH	RH
1	-3.56	-4.72	1.35	-2.09	-0.70	-0.66
2	8.07	-6.39	9.69	-3.06	1.65	-2.77
3	10.05	2.48	14.29	1.02	-0.30	2.91
4	-3.75	3.33	0.54	1.07	1.38	2.17
5	2.87	15.24	3.69	12.94	1.42	3.93
6	15.32	4.97	14.77	2.50	6.02	-0.13
7	2.86	-1.47	1.23	-2.59	5.09	0.56
8	-1.36	-4.04	4.88	-4.67	-1.78	-0.01
9	-3.70	2.63	2.64	-1.41	-3.19	3.22
10	6.69	-5.96	0.71	-1.71	8.50	-2.75
11	5.24	-0.57	5.17	0.87	-2.25	-0.86
12	1.53	-5.85	5.96	-6.59	-3.63	-0.12
13	-0.00	-2.73	5.30	1.13	-0.90	-2.86
14	1.38	-4.34	2.47	-2.33	1.62	-3.55
15	-1.08	2.63	2.20	-3.96	1.22	6.36
16	5.05	24.28	8.28	14.08	-1.64	9.96
17	11.40	0.87	17.08	-4.27	-3.97	7.82
18	9.33	6.97	7.91	3.80	3.48	2.40
19	19.31	-0.97	12.21	-2.50	8.56	2.55
20	10.89	-2.55	6.66	0.43	3.76	-0.80
21	5.96	-2.97	11.98	4.17	-4.44	-5.35
22	8.19	11.28	10.42	6.18	-1.51	0.50
23	-0.97	-1.00	6.43	2.46	-4.29	-3.65
24	7.43	3.47	6.59	2.75	2.03	0.66
25	2.68	-10.44	9.54	-7.72	-4.39	-2.59
26	1.62	-10.70	4.43	-9.93	-0.14	-2.96
27	-5.80	-3.99	-0.58	-2.60	-4.86	0.09
28	-0.83	-2.25	6.10	-1.70	-4.78	-1.75
29	-10.11	3.30	-1.70	5.84	-4.64	-3.84
30	0.85	-10.53	-2.44	-2.12	3.44	-8.50

Table S7: Details of harmonic potentials employed in each REUS window for folding free energy calculations, specifying the positions of the centers (r_0 [nm]) and the spring constants (κ [kJ/(mol·nm^2)]).

Window:		1	2	3	4	5	6	7	8	9	10	11	12	13	14	15	16	17	18
RH dimer	r_0	0.36	0.38	0.41	0.44	0.5	0.57	0.66	0.75	0.84	0.93	1.02	1.11	1.2	1.29	1.38	1.47	–	–
	κ	2.5	2.5	2.5	2.5	2.5	2.5	2.5	2.5	2.5	2.5	2.5	2.5	2.5	2.5	2.5	2.5	–	–
LH dimer	r_0	0.36	0.38	0.4	0.43	0.46	0.5	0.56	0.62	0.69	0.76	0.85	0.95	1.05	1.15	1.26	1.38	–	–
	κ	2.5	2.5	2.5	2.5	2.5	2.5	2.5	2.5	2.5	2.5	2.5	2.5	2.5	2.5	2.5	2.5	–	–
RH(−)T1	r_0	0.36	0.38	0.4	0.42	0.44	0.5	0.56	0.62	0.69	0.76	0.84	0.92	1.0	1.08	1.16	1.24	1.32	1.4
	κ	2.5	2.5	2.5	2.5	2.5	2.5	2.5	2.5	2.5	2.5	2.5	2.5	2.5	2.5	2.5	2.5	2.5	2.5
RH(−)T2	r_0	0.36	0.39	0.42	0.46	0.5	0.56	0.62	0.68	0.75	0.82	0.9	0.98	1.06	1.14	1.22	1.3	1.38	1.46
	κ	2.5	2.5	2.5	2.5	2.5	2.5	2.5	2.5	2.5	2.5	2.5	2.5	2.5	2.5	2.5	2.5	2.5	2.5
RH(−)T3	r_0	0.36	0.39	0.42	0.46	0.5	0.56	0.6	0.64	0.68	0.72	0.8	0.88	0.97	1.06	1.15	1.24	1.34	1.44
	κ	2.5	2.5	2.5	2.5	2.5	2.5	2.5	2.5	2.5	2.5	2.5	2.5	2.5	2.5	2.5	2.5	2.5	2.5
LH(+)T1	r_0	0.36	0.38	0.41	0.44	0.5	0.57	0.62	0.66	0.7	0.76	0.86	0.96	1.07	1.18	1.29	1.4	–	–
	κ	2.5	2.5	2.5	2.5	2.5	2.5	5.0	2.5	5.0	2.5	2.5	2.5	2.5	2.5	2.5	2.5	–	–
LH(+)T2	r_0	0.35	0.36	0.38	0.395	0.41	0.44	0.5	0.57	0.62	0.66	0.7	0.76	0.86	0.96	1.07	1.18	1.29	1.4
	κ	3.5	2.5	2.5	3.5	2.5	2.5	2.5	2.5	4.0	2.5	4.0	2.5	2.5	2.5	2.5	2.5	2.5	2.5
LH(+)T3	r_0	0.35	0.36	0.38	0.395	0.41	0.44	0.5	0.57	0.62	0.66	0.7	0.76	0.86	0.96	1.07	1.18	1.29	1.4
	κ	4.0	2.5	2.5	4.0	2.5	2.5	2.5	2.5	4.0	2.5	4.0	2.5	2.5	2.5	2.5	2.5	2.5	2.5

Table S8: Details of harmonic potentials employed in each REUS window for calculating the free energy profile for the transition of the GpG dinucleotide between the LH and RH conformations, specifying the positions of the centers (r_0 [nm]) and the spring constants (κ [kJ/(mol·nm^2)]).

Window:		1	2	3	4	5	6	7	8	9	10
GpG	r_0	-0.18	-0.14	-0.1	-0.06	-0.02	0.02	0.06	0.1	0.14	0.18
	κ	8.0	6.0	5.0	5.0	5.0	5.0	5.0	5.0	6.0	8.0

Table S9: Positions of the centers (r_0 [nm]) and spring constants (κ [kJ/(mol·nm^2)]) of the harmonic potentials used in each REUS window for computing the free energy landscapes governing the transition between the LH and RH conformation for GGTGG and GGTTTGG DNA oligonucleotides.

Window:		1	2	3	4	5	6	7	8	9	10	11	12	13	14	15	16
(−)T1	r_0	-0.4	-0.36	-0.32	-0.27	-0.21	-0.15	-0.09	-0.03	0.03	0.09	0.15	0.21	0.27	0.32	0.36	0.4
	κ	27.0	24.0	20.0	17.0	14.0	10.0	7.0	5.0	5.0	5.0	5.0	5.0	5.0	5.0	7.0	9.0
(−)T3	r_0	-0.4	-0.36	-0.32	-0.27	-0.21	-0.15	-0.09	-0.03	0.03	0.09	0.15	0.21	0.27	0.32	0.36	0.4
	κ	5.0	5.0	5.0	5.0	5.0	5.0	5.0	5.0	5.0	5.0	5.0	5.0	5.0	5.0	5.0	5.0
(+)T1	r_0	-0.4	-0.36	-0.32	-0.27	-0.21	-0.15	-0.09	-0.03	0.03	0.09	0.15	0.21	0.27	0.32	0.36	0.4
	κ	10.0	8.0	7.0	5.0	5.0	5.0	5.0	5.0	5.0	5.0	8.0	11.0	15.0	18.0	22.0	25.0
(+)T3	r_0	-0.4	-0.36	-0.32	-0.27	-0.21	-0.15	-0.09	-0.03	0.03	0.09	0.15	0.21	0.27	0.32	0.36	0.4
	κ	5.0	5.0	5.0	5.0	5.0	5.0	5.0	5.0	5.0	5.0	5.0	5.0	5.0	5.0	5.0	5.0

Table S10: Details of harmonic potentials employed in each REUS window for calculating the free energy profile for the transition between LH and RH helicity of the complete G4 structures, specifying the positions of the centers (r_0 [nm]) and the spring constants (κ [kJ/(mol·nm^2)]).

Window:		1	2	3	4	5	6	7	8	9	10	11	12	13	14	15	16	17
(−)T1	r_0	-0.28	-0.26	-0.24	-0.22	-0.19	-0.16	-0.12	-0.08	-0.04	0.0	0.04	0.08	0.12	0.16	0.2	0.23	0.26
	κ	35.0	25.0	21.0	17.0	15.0	13.0	10.0	10.0	10.0	10.0	10.0	10.0	10.0	10.0	10.0	10.0	10.0
(−)T3	r_0	-0.26	-0.24	-0.22	-0.2	-0.18	-0.15	-0.12	-0.08	-0.04	0.0	0.04	0.08	0.12	0.16	0.2	0.24	0.28
	κ	30.0	30.0	25.0	22.0	18.0	14.0	10.0	10.0	10.0	10.0	10.0	10.0	10.0	10.0	10.0	10.0	10.0
(+)T1	r_0	-0.28	-0.24	-0.2	-0.16	-0.12	-0.08	-0.04	0.0	0.04	0.08	0.12	0.15	0.18	0.21	0.24	0.26	0.28
	κ	10.0	10.0	10.0	10.0	10.0	10.0	10.0	10.0	10.0	10.0	10.0	10.0	15.0	15.0	17.0	20.0	25.0
(+)T3	r_0	-0.28	-0.24	-0.2	-0.16	-0.12	-0.08	-0.04	0.0	0.04	0.08	0.12	0.15	0.18	0.21	0.24	0.26	0.28
	κ	10.0	10.0	10.0	10.0	10.0	10.0	10.0	10.0	10.0	10.0	10.0	10.0	10.0	10.0	10.0	10.0	10.0

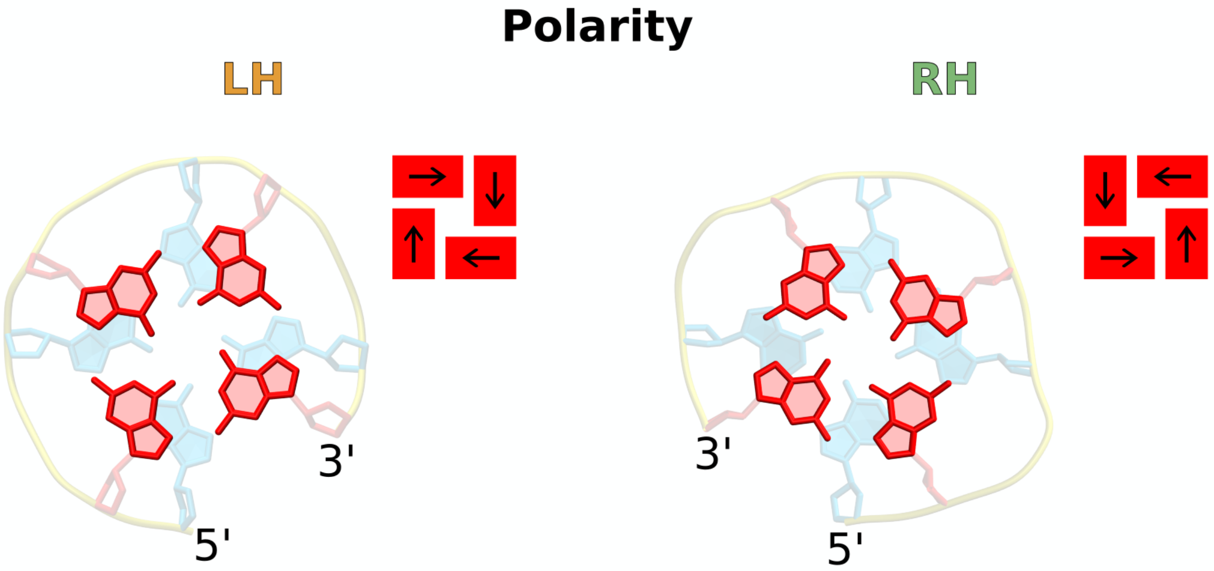

Figure S1: Different directions of strand progression typically observed for LH and RH G4s, in combination with the same *anti* glycosidic conformation, lead to opposing polarities of the G-tetrads in both cases.

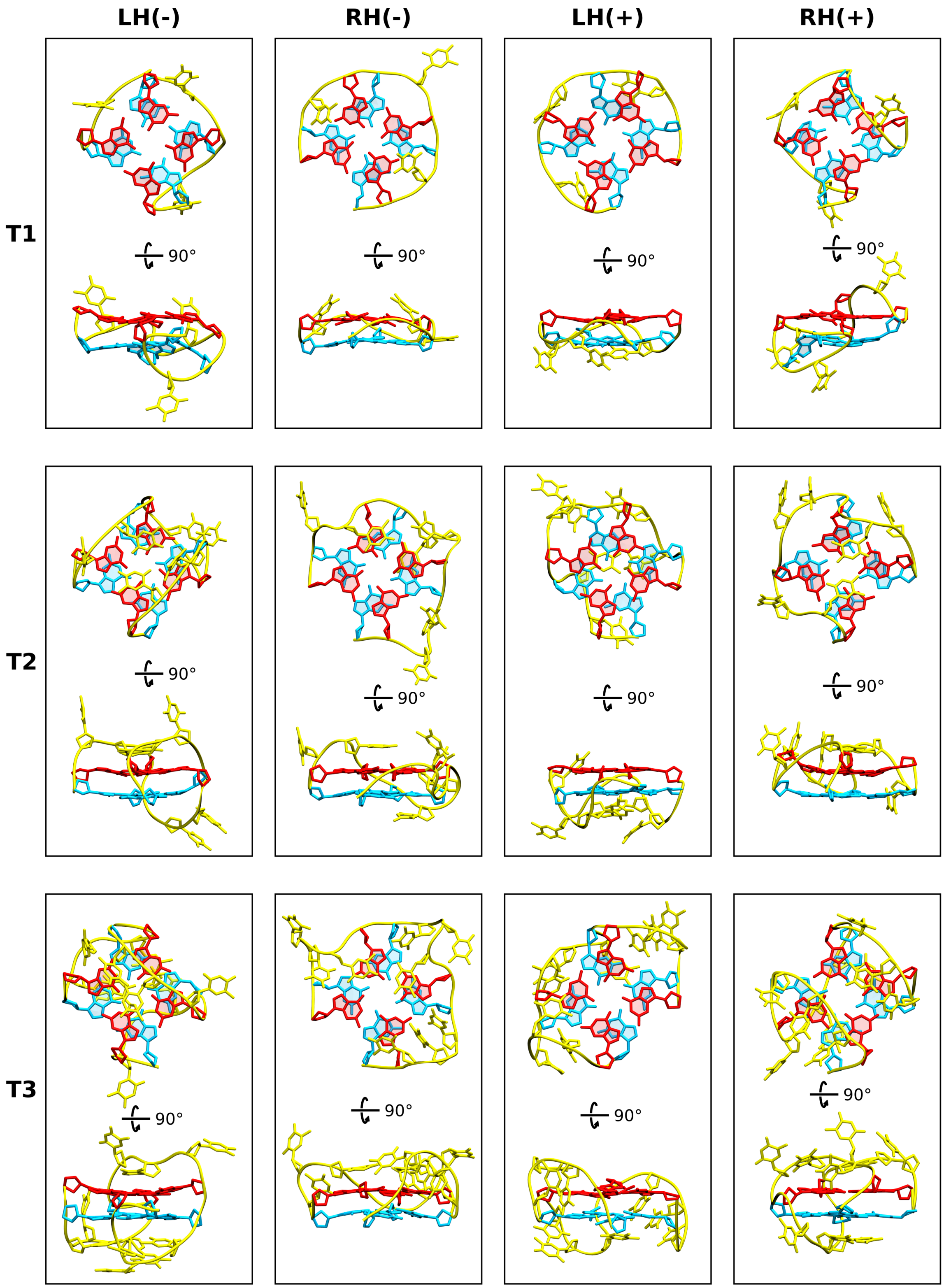

Figure S2: Initial folded configurations of G4s used in our unbiased simulations of a systematic set of G4 structures involving two different helicities (LH and RH), two opposing directions of strand progression [(+), (−)] and three distinct loop lengths (T1–T3).

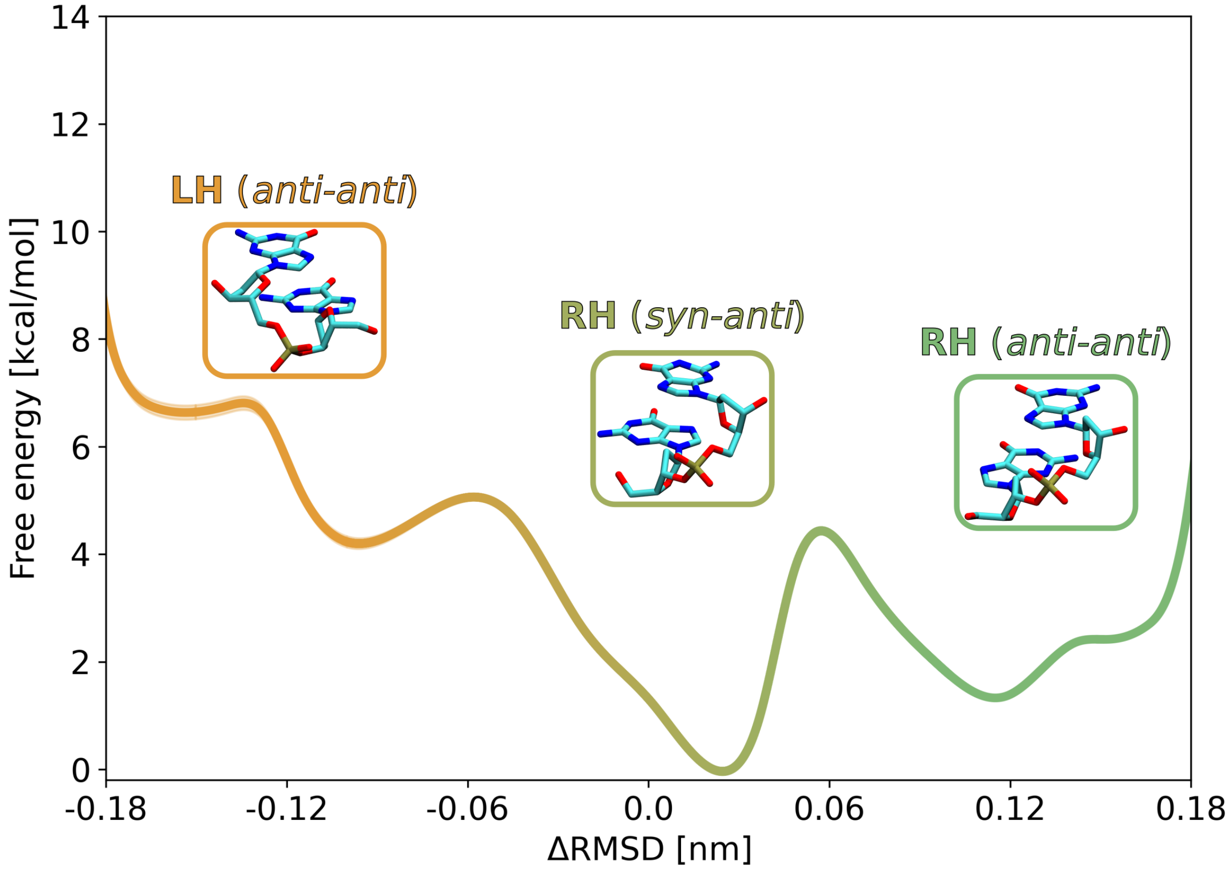

Figure S3: Free energy profile for the transition of a guanine dinucleotide (G-tract) between the LH and RH helicity, calculated using the Amber OL15 force field. The reaction coordinate is defined as the difference in root mean square deviation (RMSD) from the LH and RH G-tract conformations extracted from the full G4s.

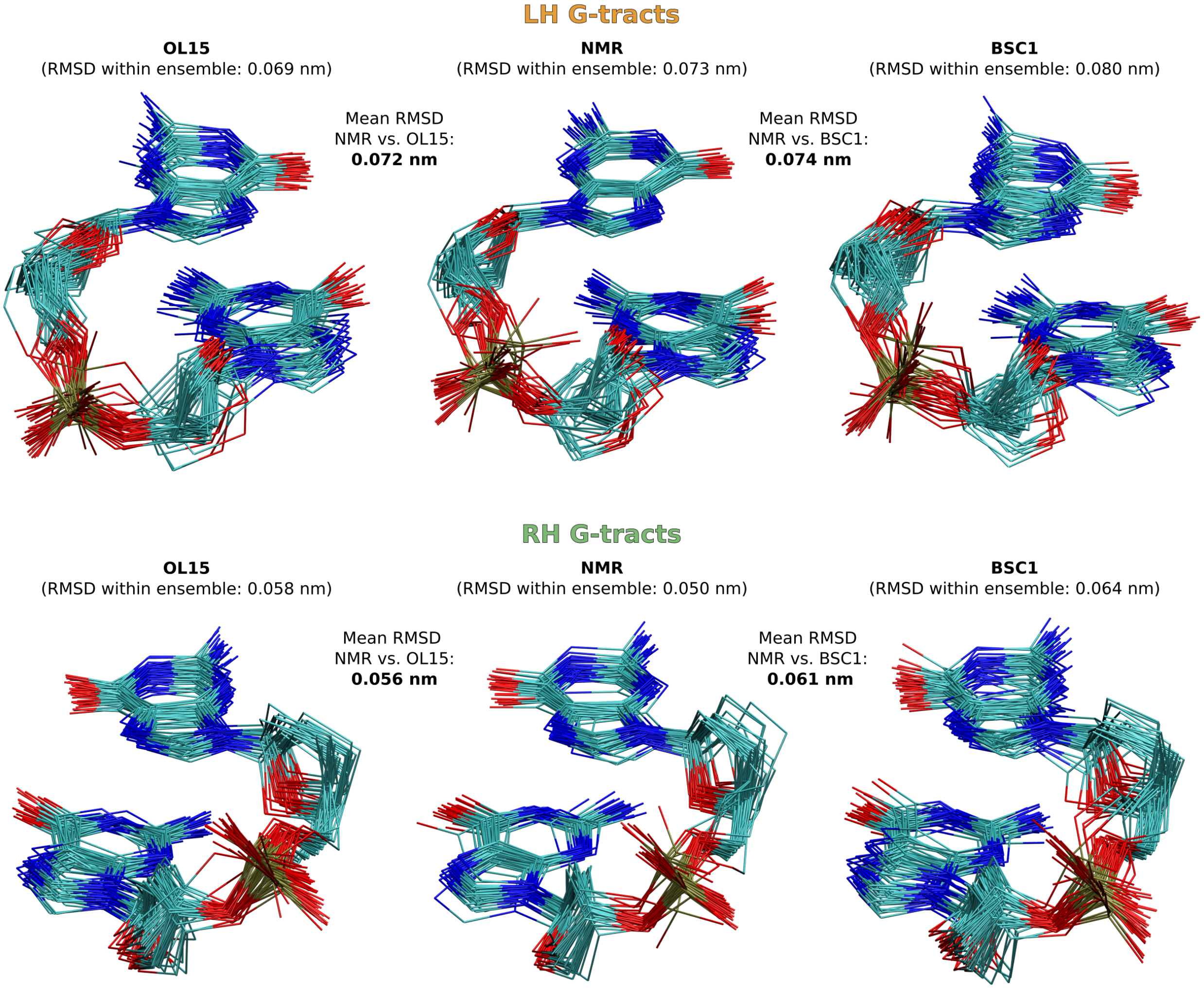

Figure S4: Evaluation of the LH and RH G-tract conformations sampled from MD trajectories of full G-quadruplexes against the NMR data. The structural ensembles presented consist of 40 conformations (10 per G-tract) extracted either from MD simulations, performed using Amber bsc1 and OL15, or from 10 models representing the NMR G-quadruplex structures (the 5'-end G4 block in 2MS9 and the 3'-end block in 2N3M were used for LH and RH helicities, respectively). The conformations were superposed by minimizing the heavy-atom RMSD, excluding the terminal C5', O5' and O3' atoms. Intra-ensemble RMSD values were computed as the mean RMSD for each conformation pair within the ensemble. Inter-ensemble RMSD values between NMR and MD-generated ensembles were calculated as the mean RMSD for each conformation pair across the ensembles.

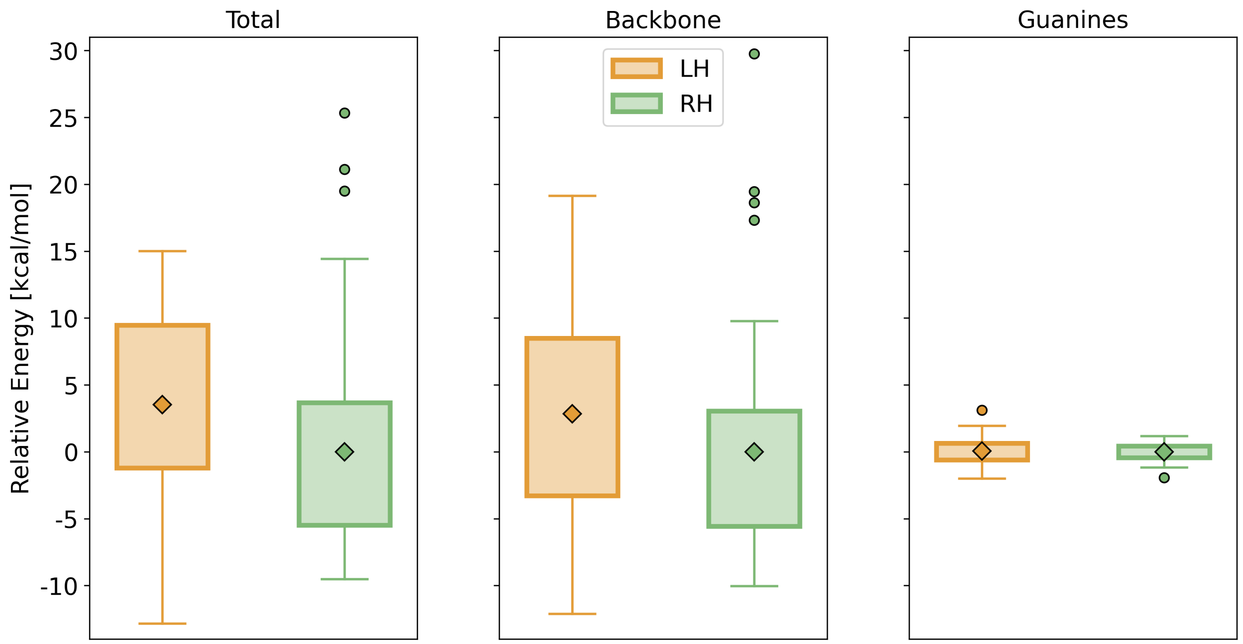

Figure S5: Guanine dinucleotide (G-tract) energies in the LH and RH conformations computed at the SCS-MP2 level for a set of 40 configurations extracted from LH and RH NMR structures (5'-end block from 2MS9 and 3'-end block from 2N3M respectively). The total energies are decomposed approximately into the intrinsic energy of the sugar-phosphate backbone and the energy of guanine-guanine interaction.

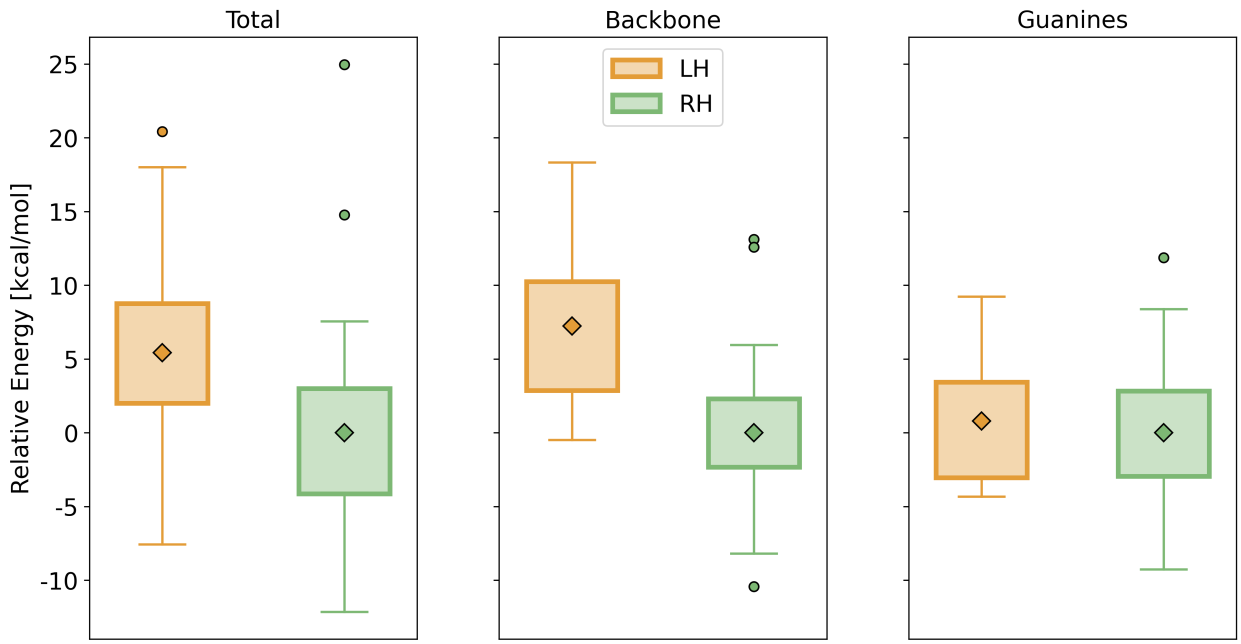

Figure S6: Guanine dinucleotide (G-tract) energies in the LH and RH conformations computed at the DFT level for a set of 30 configurations extracted from our MD-generated ensembles of both states (using Amber bsc1). The total energies are decomposed approximately into the intrinsic energy of the sugar-phosphate backbone and the energy of guanine-guanine interaction. For detailed energies consult Table S5.

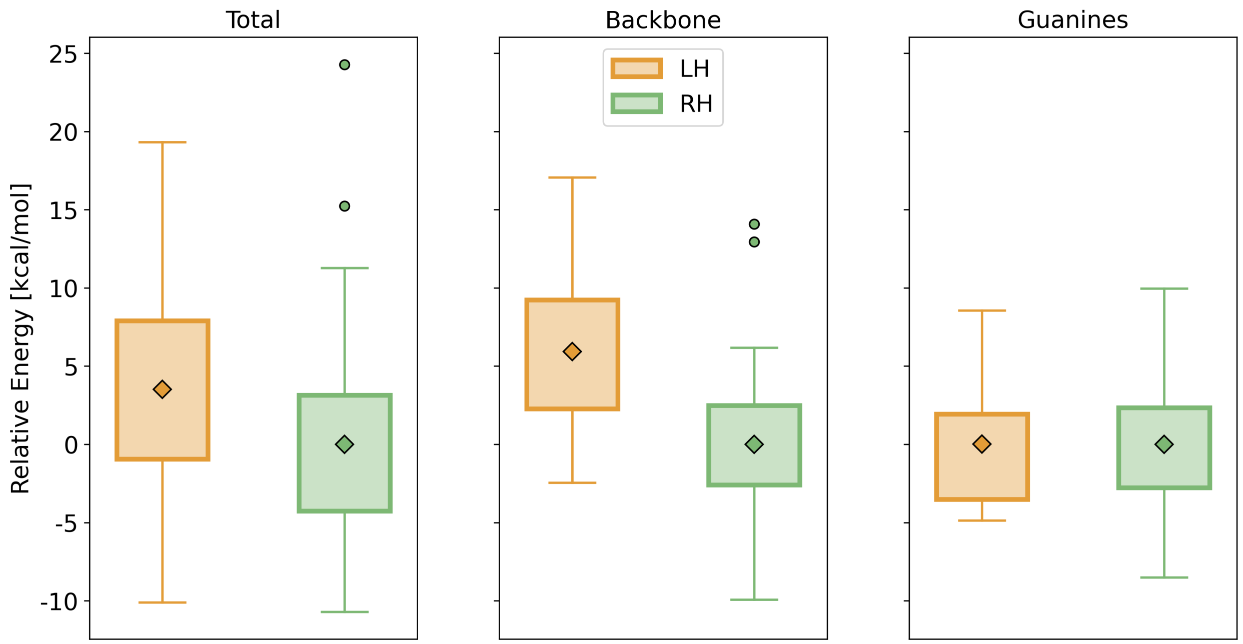

Figure S7: Guanine dinucleotide (G-tract) energies in the LH and RH conformations computed at the SCS-MP2 level for a set of 30 configurations extracted from our MD-generated ensembles of both states (using Amber bsc1). The total energies are decomposed approximately into the intrinsic energy of the sugar-phosphate backbone and the energy of guanine-guanine interaction. For detailed energies consult Table S6.

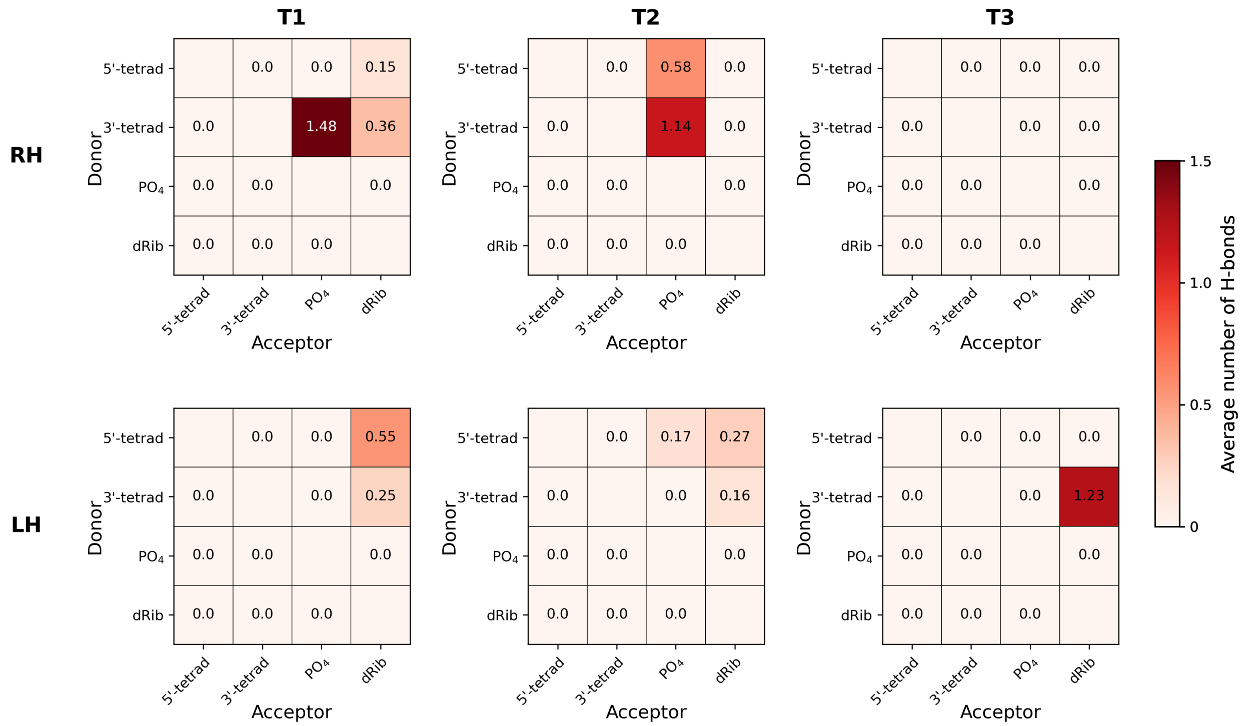

Figure S8: Average count of intramolecular hydrogen bonds formed between specific structural elements of G4 structures, computed based on the MD trajectories of the fully-folded LH(+) and RH(−) G4s with three different loop lengths (T1–T3). '3′-tetrad' and '5′'-tetrad denote the guanines in the 3′-terminal- and 5′-terminal G-tetrad, respectively, 'PO_4' stand for the phosphate groups and 'dRib' for the deoxyribose residues. The following geometric criteria for hydrogen bonds were used: a hydrogen–acceptor distance of less than 0.25 nm and a donor–hydrogen–acceptor angle ranging from 145 to 180°.

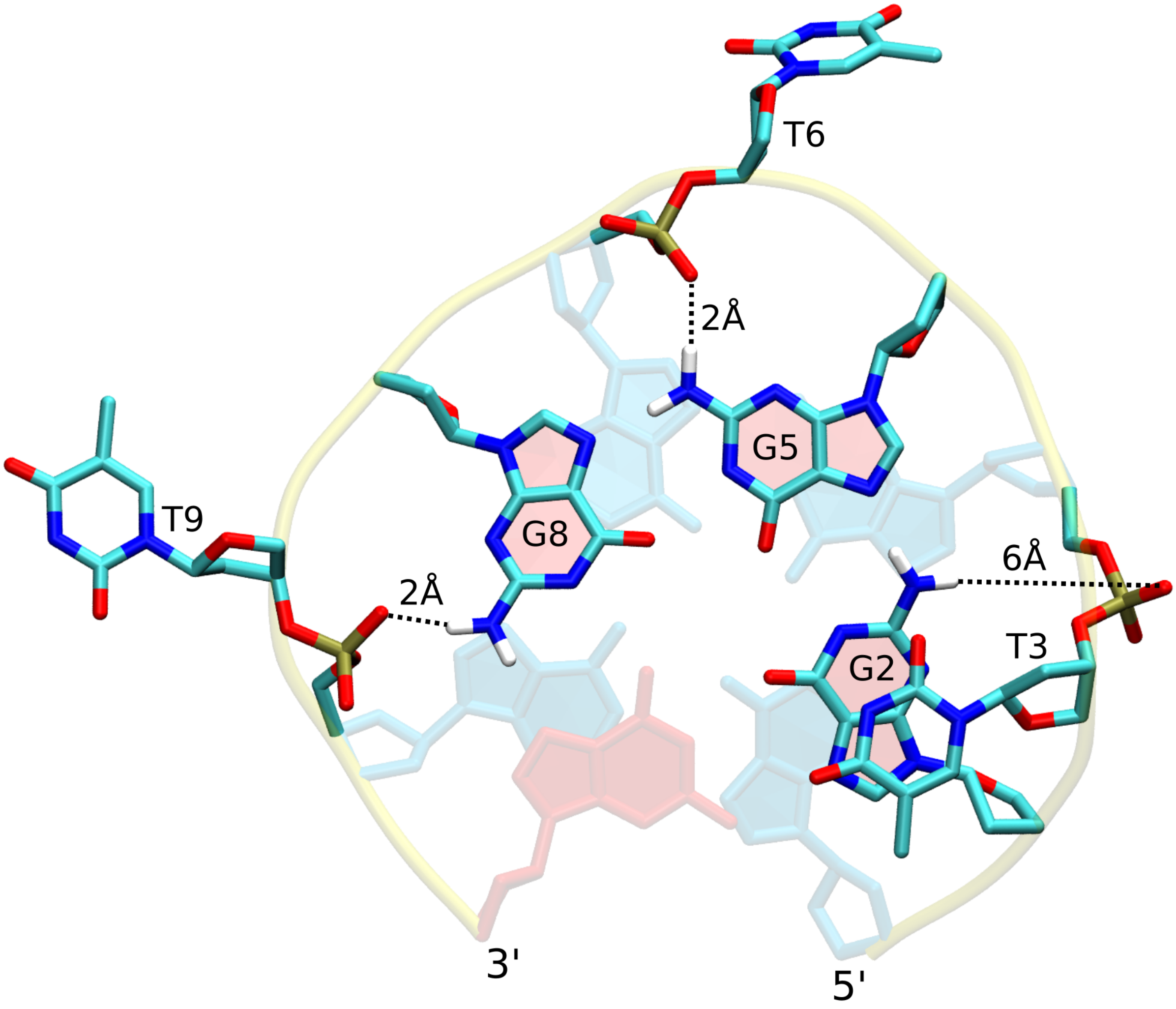

Figure S9: Comparison of the 'stacked' (T3) and 'exposed' (T6 and T9) conformations of the loop thymines in the right-handed all-parallel G4 viewed from the top.

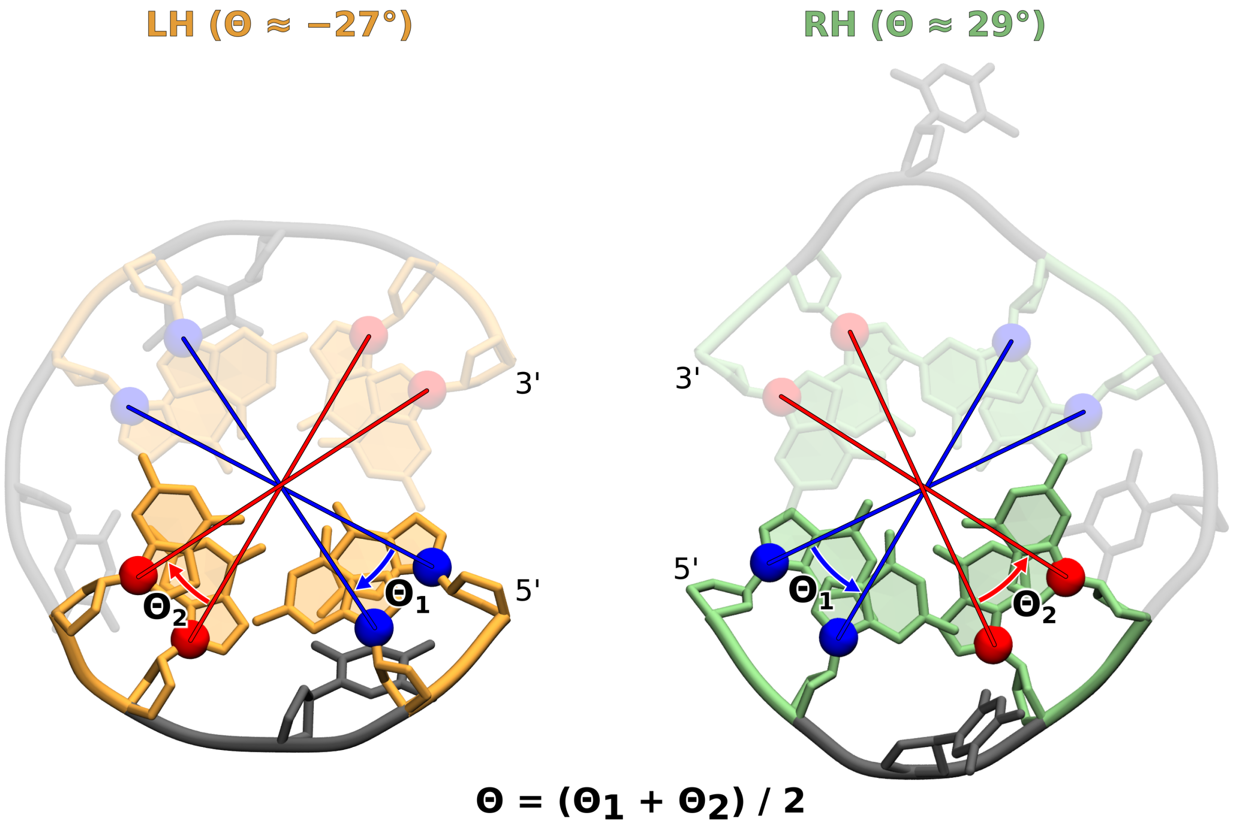

Figure S10: Definition of the twist angle Θ used to study the transition between the left- and right-handed helicity. Θ is defined as and arithmetic average of Θ_1 and Θ_2, where the latter are angles between the vectors connecting the N9 atoms (shown as spheres) in guanines located diagonally in each of the tetrads.

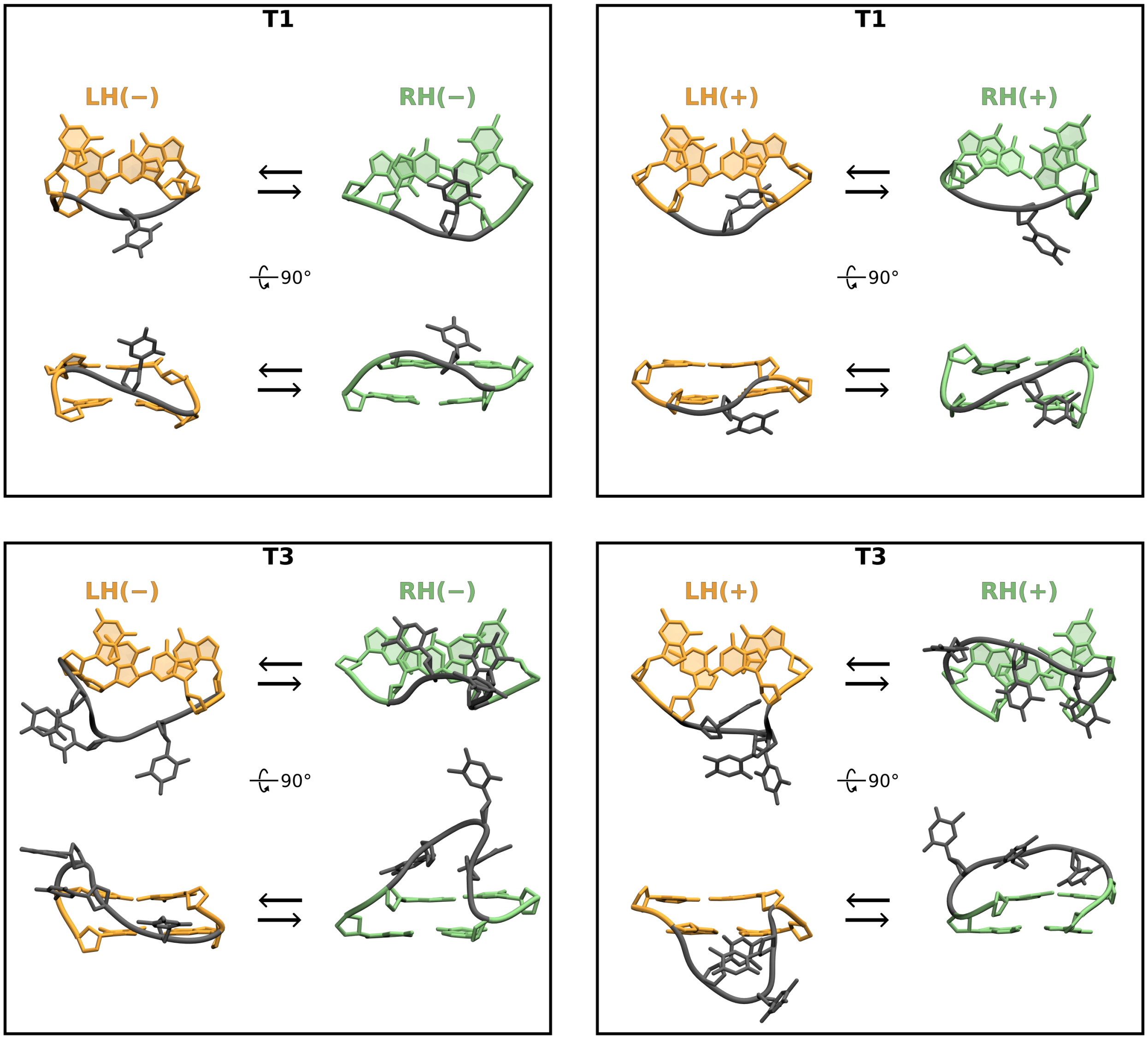

Figure S11: All four conformational transitions considered when investigating the relation between the helicity and strand progression using two model DNA fragments differing in the loop length (T1 and T3). All the reference conformations of the model DNA fragments were extracted from the appropriate full G4s.

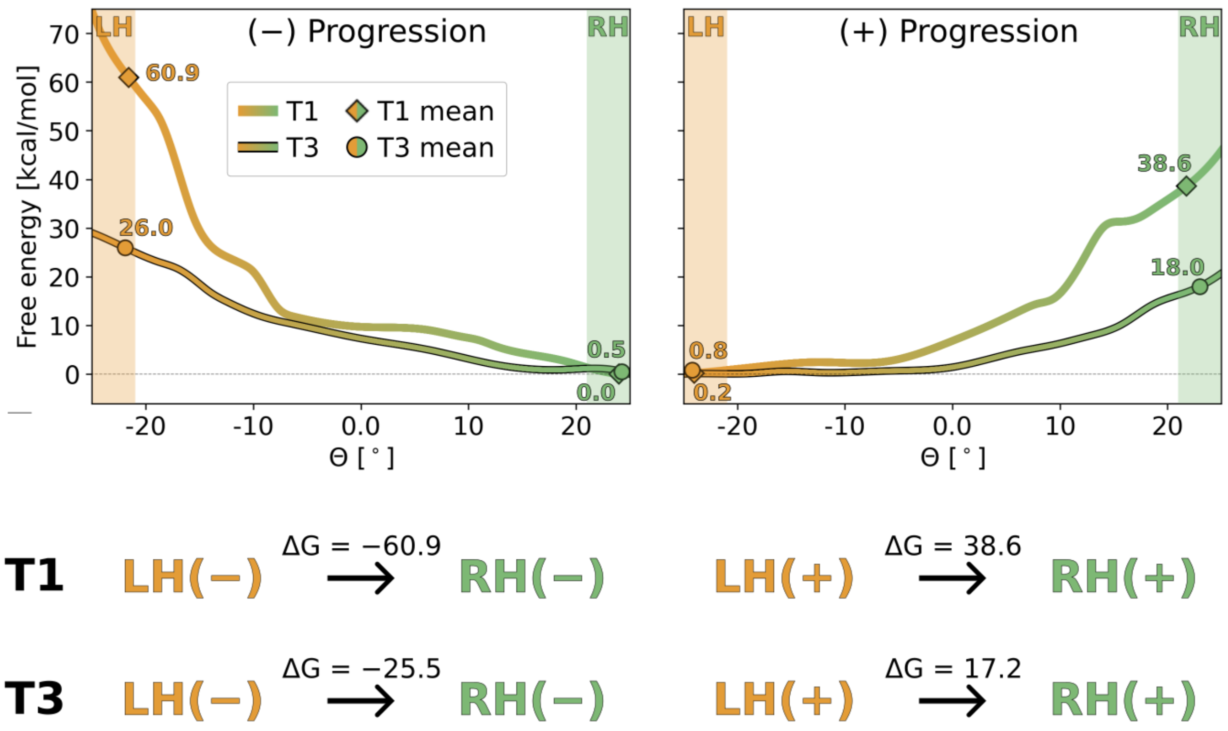

Figure S12: Free energy profiles for the transition of the all-parallel G4 between the LH and RH helicity, determined separately for the (−) and (+) strand progression. Two different loop lengths were condsidered (T1 and T3). The corresponding free energy differences between the LH and RH states are given below (in kcal/mol).

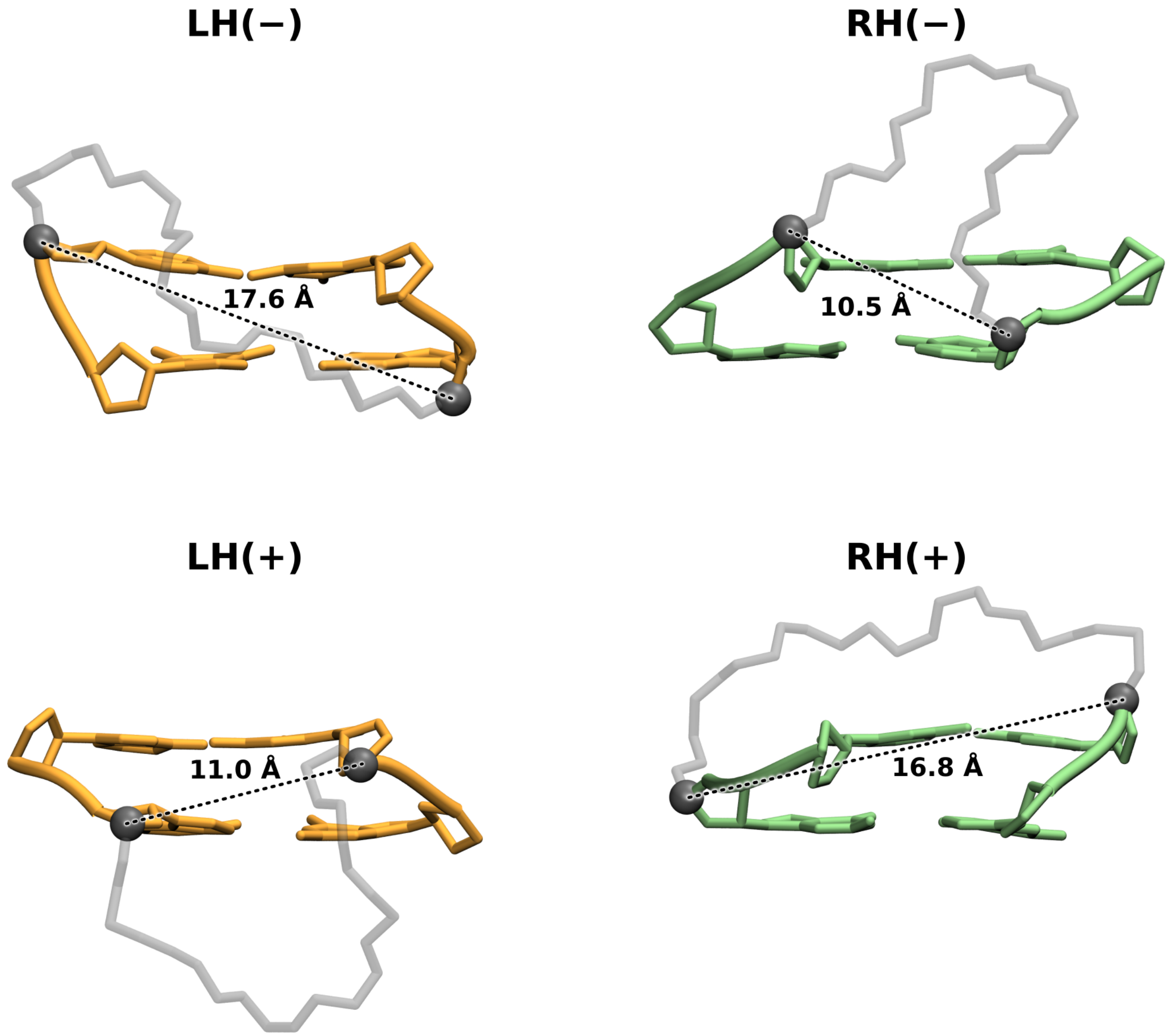

Figure S13: Average distances between the loop attachment points in the RH and LH states for (−) and (+) directions of strand progression.

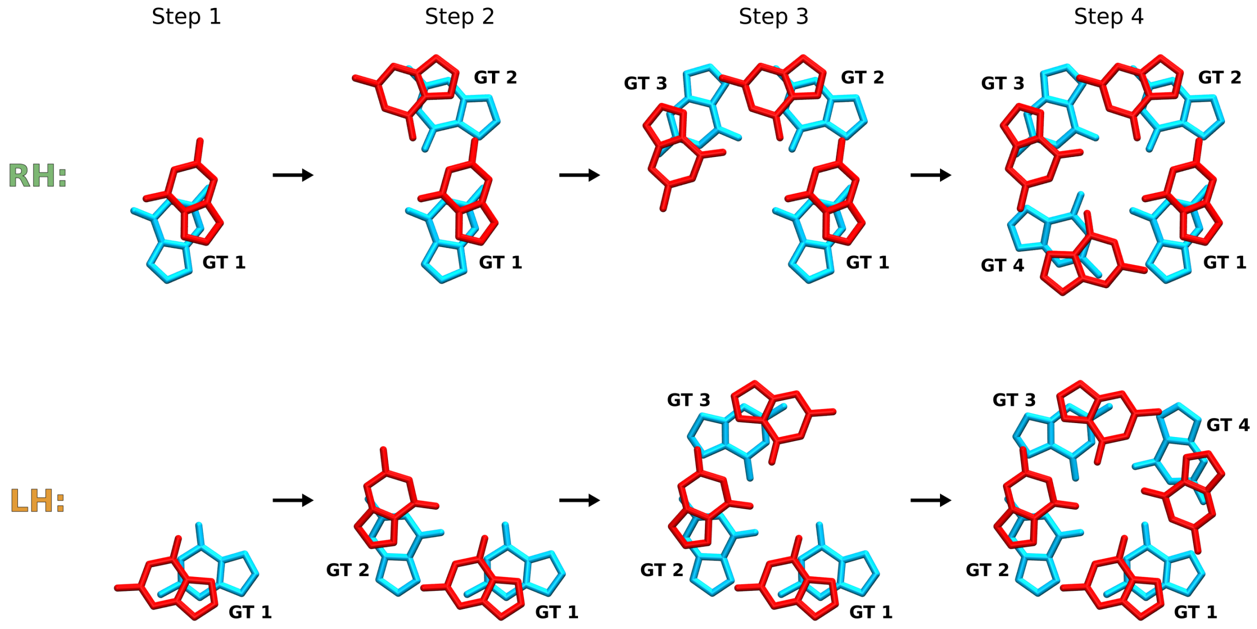

Figure S14: Reference structures of guanine cores used in our steered-MD-based *de novo* folding procedure. The G-quadruplex (G4) formation follows a stepwise approach utilizing four reference structures, each incorporating one additional guanine pair compared to the previous one. This stepwise assembly allows for the complete G4 formation in a G-tract by G-tract manner (GT1→GT2→GT3→GT4). The references, which include only guanine heavy atoms, were extracted from the NMR structures of the $(TGG)_4$ block (PDB id: 2N3M) and the $(GGT)_4$ block (PDB id: 2MS9) for the RH and LH G4 structures, respectively.

a

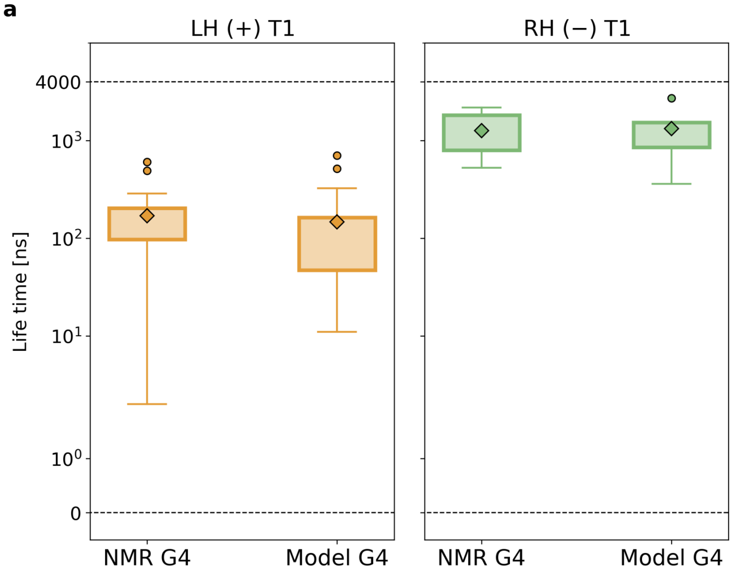

b

	LH (+) T1		RH (−) T1	
	NMR G4	Model G4	NMR G4	Model G4
1	117.0	122.0	526.0	853.0
2	60.0	177.0	997.0	360.0
3	197.0	20.0	1817.0	1535.0
4	141.0	11.0	796.0	2722.0
5	491.0	98.0	2185.0	1184.0
6	93.0	48.0	-	-
7	205.0	515.0	-	-
8	2.0	65.0	-	-
9	109.0	73.0	-	-
10	133.0	13.0	-	-
11	286.0	30.0	-	-
12	604.0	182.0	-	-
13	6.0	51.0	-	-
14	142.0	91.0	-	-
15	130.0	702.0	-	-
16	110.0	47.0	-	-
17	31.0	325.0	-	-
18	210.0	77.0	-	-

Figure S15: (**a**) Comparison of the folded-state lifetimes of LH(+) and RH(−) G4 structures with T1 loops obtained via our *de novo* folding procedure (Model G4) and those determined by NMR spectroscopy (NMR G4). (**b**) Detailed folded-state lifetimes obtained from all individual MD simulations of the considered systems.

Movie S1: Steered-MD-based folding simulations for RH(−) and LH(+) structures with T1 loops. As the folding proceeds, the consecutive segments of the DNA strand are being colored such that the colored part corresponds to the appropriate core reference in a given step (see Fig. S10). 5'-tetrad is presented in cyan, 3'-tetrad in red, and the backbone and thymines in yellow, consistently with Fig. 1. The movie was prepared using VMD[26] and the Molywood package.[27]

References

(1) Jorgensen, W. L.; Chandrasekhar, J.; Madura, J. D.; Impey, R. W.; Klein, M. L. Comparison of simple potential functions for simulating liquid water. *J. Chem. Phys.* **1983**, *79*, 926–935.

(2) Ivani, I.; Dans, P. D.; Noy, A.; Pérez, A.; Faustino, I.; Hospital, A.; Walther, J.; Andrio, P.; Goñi, R.; Balaceanu, A., et al. Parmbsc1: a refined force field for DNA simulations. *Nat. Methods* **2016**, *13*, 55–58.

(3) Joung, I. S.; Cheatham III, T. E. Determination of alkali and halide monovalent ion parameters for use in explicitly solvated biomolecular simulations. *J. Phys. Chem. B* **2008**, *112*, 9020–9041.

(4) Chung, W. J.; Heddi, B.; Schmitt, E.; Lim, K. W.; Mechulam, Y.; Phan, A. T. Structure of a left-handed DNA G-quadruplex. *Proc. Natl. Acad. Sci. U. S. A.* **2015**, *112*, 2729–2733.

(5) Do, N. Q.; Chung, W. J.; Truong, T. H. A.; Heddi, B.; Phan, A. T. G-quadruplex structure of an anti-proliferative DNA sequence. *Nucleic Acids Res.* **2017**, *45*, 7487–7493.

(6) Abraham, M. J.; Murtola, T.; Schulz, R.; Páll, S.; Smith, J. C.; Hess, B.; Lindahl, E. GROMACS: High performance molecular simulations through multi-level parallelism from laptops to supercomputers. *SoftwareX* **2015**, *1*, 19–25.

(7) Tribello, G. A.; Bonomi, M.; Branduardi, D.; Camilloni, C.; Bussi, G. PLUMED 2: New feathers for an old bird. *Comput. Phys. Commun.* **2014**, *185*, 604–613.

(8) Bussi, G.; Donadio, D.; Parrinello, M. Canonical sampling through velocity rescaling. *J. Chem. Phys.* **2007**, *126*.

(9) Parrinello, M.; Rahman, A. Polymorphic transitions in single crystals: A new molecular dynamics method. *J. Appl. Phys.* **1981**, *52*, 7182–7190.

(10) Darden, T.; York, D.; Pedersen, L. Particle mesh Ewald: An N· log (N) method for Ewald sums in large systems. *J. Chem. Phys.* **1993**, *98*, 10089–10092.

(11) Hess, B. P-LINCS: A parallel linear constraint solver for molecular simulation. *J. Chem. Theory Comput.* **2008**, *4*, 116–122.

(12) Miyamoto, S.; Kollman, P. A. Settle: An analytical version of the SHAKE and RATTLE algorithm for rigid water models. *J. Comput. Chem.* **1992**, *13*, 952–962.

(13) Torrie, G. M.; Valleau, J. P. Nonphysical sampling distributions in Monte Carlo free-energy estimation: Umbrella sampling. *J. Comput. Phys.* **1977**, *23*, 187–199.

(14) Kumar, S.; Rosenberg, J. M.; Bouzida, D.; Swendsen, R. H.; Kollman, P. A. Multidimensional free-energy calculations using the weighted histogram analysis method. *J. Comput. Chem.* **1995**, *16*, 1339–1350.

(15) Galindo-Murillo, R.; Robertson, J. C.; Zgarbova, M.; Sponer, J.; Otyepka, M.; Jurecka, P.; Cheatham III, T. E. Assessing the current state of amber force field modifications for DNA. *J. Chem. Theory Comput.* **2016**, *12*, 4114–4127.

(16) Neese, F. Software update: The ORCA program system—Version 5.0. *WIREs Comput Mol Sci* **2022**, *12*, e1606.

(17) Becke, A. D. Density-functional thermochemistry. I. The effect of the exchange-only gradient correction. *J. Chem. Phys.* **1992**, *96*, 2155–2160.

(18) Grimme, S. Density functional theory with London dispersion corrections. *WIREs Comput Mol Sci* **2011**, *1*, 211–228.

(19) Krishnan, R.; Binkley, J. S.; Seeger, R.; Pople, J. A. Self-consistent molecular orbital methods. XX. A basis set for correlated wave functions. *J. Chem. Phys.* **1980**, *72*, 650–654.

(20) Head-Gordon, M.; Pople, J. A.; Frisch, M. J. MP2 energy evaluation by direct methods. *Chem. Phys. Lett.* **1988**, *153*, 503–506.

(21) Kendall, R. A.; Dunning Jr, T. H.; Harrison, R. J. Electron affinities of the first-row atoms revisited. Systematic basis sets and wave functions. *J. Chem. Phys.* **1992**, *96*, 6796–6806.

(22) Bakalar, B.; Heddi, B.; Schmitt, E.; Mechulam, Y.; Phan, A. T. A Minimal Sequence for Left-Handed G-Quadruplex Formation. *Angew. Chem. Int. Ed.* **2019**, *58*, 2331–2335.

(23) Winnerdy, F. R.; Bakalar, B.; Maity, A.; Vandana, J. J.; Mechulam, Y.; Schmitt, E.; Phan, A. T. NMR solution and X-ray crystal structures of a DNA molecule containing both right-and left-handed parallel-stranded G-quadruplexes. *Nucleic Acids Res.* **2019**, *47*, 8272–8281.

(24) Das, P.; Ngo, K. H.; Winnerdy, F. R.; Maity, A.; Bakalar, B.; Mechulam, Y.; Schmitt, E.; Phan, A. T. Bulges in left-handed G-quadruplexes. *Nucleic Acids Res.* **2021**, *49*, 1724–1736.

(25) Das, P.; Winnerdy, F. R.; Maity, A.; Mechulam, Y.; Phan, A. T. A novel minimal motif for left-handed G-quadruplex formation. *Chem. Commun.* **2021**, *57*, 2527–2530.

(26) Humphrey, W.; Dalke, A.; Schulten, K. VMD: visual molecular dynamics. *J. Mol. Graphics* **1996**, *14*, 33–38.

(27) Wieczór, M.; Hospital, A.; Bayarri, G.; Czub, J.; Orozco, M. Molywood: streamlining the design and rendering of molecular movies. *Bioinformatics* **2020**, *36*, 4660–4661.
